# Comparability of Heart Rate Turbulence Methodology: 15 Intervals Suffice to Calculate Turbulence Slope – A Methodological Analysis Using PhysioNet Data of 1074 Patients

**DOI:** 10.3389/fcvm.2022.793535

**Published:** 2022-04-06

**Authors:** Valeria Blesius, Christopher Schölzel, Gernot Ernst, Andreas Dominik

**Affiliations:** ^1^Life Science Informatics Group, Department of Mathematics, Natural Sciences and Informatics, Technische Hochschule Mittelhessen (THM) University of Applied Sciences, Giessen, Germany; ^2^Department of Anaesthesiology, Kongsberg Hospital, Vestre Viken Hospital Trust, Kongsberg, Norway; ^3^Psychological Institute, University of Oslo, Oslo, Norway

**Keywords:** heart rate turbulence (HRT), noninvasive risk stratification, heart rate variability (HRV), heart failure, myocardial infarction, methodology, standardization

## Abstract

Heart rate turbulence (HRT) is a characteristic heart rate pattern triggered by a ventricular premature contraction (VPC). It can be used to assess autonomic function and health risk for various conditions, e.g., coronary artery disease or cardiomyopathy. While comparability is essential for scientific analysis, especially for research focusing on clinical application, the methodology of HRT still varies widely in the literature. Particularly, the ECG measurement and parameter calculation of HRT differs, including the calculation of turbulence slope (TS). In this article, we focus on common variations in the number of intervals after the VPC that are used to calculate TS (#TSRR) posing two questions: 1) Does a change in #TSRR introduce noticeable changes in HRT parameter values and classification? and 2) Do larger values of turbulence timing (TT) enabled by a larger #TSRR still represent distinct HRT? We compiled a free-access data set of 1,080 annotated long-term ECGs provided by Physionet. HRT parameter values and risk classes were determined both with #TSRR 15 and 20. A standard local tachogram was created by averaging the tachograms of only the files with the best heart rate variability values. The shape of this standard VPC sequence was compared to all VPC sequences grouped by their TT value using dynamic time warping (DTW) in order to identify HRT shapes. When calculated with different #TSRR, our results show only a little difference between the number of files with enough valid VPC sequences to calculate HRT (<1%) and files with different risk classes (5 and 6% for HRT0-2 and HRTA-C, respectively). In the DTW analysis, the difference between averaged sequences with a specific TT and the standard sequence increased with increasing TT. Our analysis suggests that HRT occurs in the early intervals after the VPC and TS calculated from late intervals reflects common heart rate variability rather than a distinct response to the VPC. Even though the differences in classification are marginal, this can lead to problems in clinical application and scientific research. Therefore, we recommend uniformly using #TSRR 15 in HRT analysis.

## 1. Introduction

### 1.1. Heart Rate Turbulence

With a simple point-of-care investigation of the heart rate, it is possible to estimate the condition and prognosis of patients. A possible method is HRT, which is a naturally occurring phenomenon that arises after a VPC (2): The characteristic pattern comprises an initial drop of interval length (IL) followed by slowly increasing and afterward decreasing length (refer to the Supplementary Figure 1 for a visual representation). This heart rate fluctuation is provoked by the ineffectiveness of the premature beat, which leads to a drop of blood pressure and activates the baroreflex (3, 4).

Because of this dependency on the autonomic nervous system (ANS), HRT can be used as a marker for autonomic health (5). Studies have shown that HRT parameters can be useful risk indicators for all-cause mortality after myocardial infarction or chronic heart failure (5, 6). In combination with other risk indicators, HRT can be used in clinical diagnostics to make therapeutic decisions (7–9). Several methods for the inclusion of HRT in implantable cardioverter defibrillators have already been suggested (10–12). Similarly, GE Healthcare implemented HRT assessment in their Holter analysis software tools *MARS* and *CardioDay*, which both have already been used for HRT analysis (13–15).

For HRT, there are three main parameter values that can be calculated (check the Supplementary Figure 1 for a graphical depiction): turbulence onset (TO) describes the first drop of the IL after the VPC compared to the intervals before the VPC. It is, therefore, a marker for the parasympathetic response. TS describes the steepest slope of the tachogram after the compensatory interval (compI). The third parameter TT is the index of the first interval that shows TS (4). Both TS and TT are markers for the sympathetic and parasympathetic activity.

Although the #TSRR was described in the standards as being 15 (16), many studies use 20 instead as suggested in the first description of HRT (17). The first article to give 15 as #TSRR is Barthel et al. (18) but without giving a reason for changing the original method. In reviews about HRT, there is a switch of suggesting #TSRR 20 at first (19–21) and then #TSRR 15 in recent years (2, 5, 22–25). However, many publications of late still use #TSRR 20 (26–30).

### 1.2. Rationale and Scope

Comparability is one of the key factors of scientific research, especially when developing techniques and workflows used in clinical medicine. Methodological variance diminishes comparable data and can lead to seemingly contradictory results, which make it difficult to assess the usefulness of a technique for a particular use case. For HRT, a standard methodology has been published in the “International Society for Holter and Noninvasive Electrocardiology (ISHNE)” Consensus (16). However, many studies still use different methods to assess HRT (31) causing the aforementioned difficulties.

Until now, no study has analyzed the difference in HRT parameter values when calculated from different #TSRRs. A higher #TSRR increases the risk of artifacts and other arrhythmias to lie in the required calculation range which leads to an exclusion of the VPCSs (VPC snippet, i.e., all RR intervals surrounding the VPC 78 used for HRT calculation). Conversely, with a lower #TSRR, these compromising intervals may lie outside of the needed calculation range for some VPC snippet, i.e. all RR intervals surrounding the VPC used for HRT calculations (VPCSs) which would make them shorter but valid sequences for HRT assessment. In consequence, a change in #TSRR can lead to a selection of different sets of VPCSs and, therefore, affect all HRT parameter values of a person.

Since HRT is triggered by a VPC *via* the baroreflex, it is plausible that the reaction should arise without any delay. This means that the slope that represents the turbulence should arise first in direct proximity to the compI and second always after a similar time period. Accordingly, TS calculated from either only late intervals or intervals with widely differing indices may only describe random fluctuation rather than a reaction of the ANS. Because TT describes the localization of TS, it can be used to test this assumption.

In this article, we analyze two hypotheses:

- Hypothesis 1: *There is a distinct difference in HRT parameters when calculating HRT with #TSRR 15 or 20*.We test this on a large free-access data set from Physionet and compare the resulting HRT parameters and classes.
- Hypothesis 2: *Persons with a high TT value or a high TT variability do not show HRT, but seemingly random fluctuations, i.e., heart rate variability (HRV)*.

We, therefore, create an averaged ideal standard VPCS (stVPCS) with distinct HRT by filtering the Physionet data set *via* HRV parameters. This standard VPCS is then compared with sequences that have been averaged from VPCSs sorted for their respective TT value.

## 2. Materials and Methods

### 2.1. Materials

#### 2.1.1. Data

We used databases available on physionet.org (32). The databases had to include annotations of long-term electrocardiograms (ECGs) specifying the beat types. All databases that fit those criteria at the time of analysis (15.01.2021) are summed up in Table 1.

**Table 1:** Overview of the used databases.

**ID**	**Full name**	**ECGs**	**Length**	**Corrected**	**Scope**
chfdb	BIDMC Congestive Heart Failure Database (33)	15	20	no	severe congestive heart failure (NYHA class 3-4)
chf2db	Congestive Heart Failure RR Interval Database	29	23	yes	congestive heart failure (NYHA classes 1-3)
crisdb	CAST RR Interval Sub-Study Database (34)	762	23.9	no	myocardial infarction
excluded	Recordings excluded from the NSRDB	2	22.7	unknown	low-grade arrhythmias
ltafdb	Long Term AF Database (35)	84	23.8	yes	paroxysmal or sustained atrial fibrillation
ltdb	MIT-BIH Long-Term ECG Database	7	22.2	yes	unknown
ltstdb	Long Term ST Database (36)	86	23.4	yes	variety of events of segment changes
nsrdb	MIT-BIH Normal Sinus Rhythm Database	18	21.1	unknown	no significant arrhythmias
nsr2db	Normal Sinus Rhythm RR Interval Database	54	24	yes	normal sinus rhythm
sddb	Sudden Cardiac Death Holter Database (37)	23	23.5	(yes)	sustained ventricular tachyarrhythmia, mostly with actual cardiac arrest

*The IDs correspond to the URLs under which the databases are accessible online. The number of ECGs mostly matches the number of recorded persons in each database, only for ltstdb four of the 80 persons were recorded multiple times. The columns Length and corrected give the median length of the records and whether the annotations were manually corrected, respectively. For sddb, only a part of the records had manually corrected annotations. Information that could not be found, i.e., whether annotations were corrected or the scope of the study, was marked as “unknown.” The version of all databases is 1.0.0, and they can be found on https://physionet.org/about/database/ (32)*.

Since our analysis should be independent of the medical background of measurements, we did not exclude databases based on their scope. In sum, our analysis included 1,080 annotation files. If possible, we preferred annotations that were manually corrected, although most of the databases only included automatically generated annotations.

#### 2.1.2. The RHRT Package

For the calculation of the HRT parameter values of each annotation file, we used our R package *RHRT* (v. 1.1) (38). *RHRT* provides functions to find VPCSs in time intervals and calculate HRT parameter values with customisable filtering criteria, order of calculation and normalization. The package can be found on The Comprehensive R Archive Network (CRAN) (https://CRAN.R-project.org/package=RHRT) and on github (https://github.com/VBlesius/RHRT). The default methodology of filtering, calculation, and classification is done as suggested in Blesius et al. (31), which mostly follows the ISHNE consensus (16). In contrast to the standards, we use 5 instead of 2 RR intervals in a VPCS before the couplI (preRRs), because the preceding intervals are used to calculate the reference interval (refI) and must, therefore, be included in the filtering process. Furthermore, we use TS normalized after (1) (nTS) which is TS normalized for heart rate and #TSRR. A detailed description can be found in the Supplementary Data Sheet 1 and the documentation of the package.

#### 2.1.3. Other R Packages

Statistical differences between data sets were calculated with the *stats* package (v 4.1.1). *RHRV* version 4.2.6 was used to calculate HRV parameter values. For the Poincaré filter and the data preparation of the HRV calculation, we used the packages, *geometry* (v. 0.4.5), *smoother* (v. 1.1), and *purrr* (v. 0.3.4). To compare the stVPCS with the averaged VPCSs *dtw* (v. 1.22.3) was used. This package provides functions for DTW, which is an algorithm to compare similarities of two sequences: All points of the first sequence are matched to the points of the second one whereas points can be matched to multiple other points. The only limitation is that the first and last points have to be matched to each other, respectively, and mapped indices have to be increasing, meaning that there may not be overlapping matches. Dynamic time warping (DTW) can calculate a matching score, which was used in this analysis.

### 2.2. Methods

#### 2.2.1. Comparing Data With #TSRR 15 and 20

We assessed HRT of all files twice with the default parameters of *RHRT* and the settings numPostRRs = 15 (TSRR15) and numPostRRs = 20 (TSRR20). Additionally, we created a data set from the valid VPCs included in the analysis with #TSRR 20, but calculated HRT results with numPostRRs = 15 (TSRR15∩). This leads to a data set with identical VPCs but shorter VPCSs that allows comparison without considering filtering effects. We then calculated the arithmetic mean and SD of HRT parameter values and classified the data into HRT0-2 and HRTA-C. Depending on the number of files with enough valid VPCSs, either a Welch's unequal variances *t*-test or a paired Student's *t*-test (both with *t.test* of the *stats* package) were used to detect differences between the sets of parameter values.

#### 2.2.2. Creating a stVPCS

The purpose of the analysis was to compare VPCSs sorted on the basis of TT with an stVPCS. Since HRT is most pronounced in persons without autonomic dysfunction as reviewed in Bauer et al. (16), we needed to select files of supposedly healthy persons. However, the databases used do not include data from persons labeled as healthy. It may be assumed that NSRDB and NSR2DB are comprised of such data, but manual inspection of Poincaré plots revealed abnormal patterns throughout both databases. Therefore, we used HRV to filter the files of all databases that have the most sound values of these autonomic markers. The filtering process included three steps:

##### 2.2.2.1. Length of Files

At first, all files that were shorter than 20 h or longer than 28 h were discarded. Since HRT is correlated with the circadian rhythm of the ANS, it should be calculated from measurements with a length as close as possible to 24 h or its multiple if procurable.

Applying the following filters—especially the Poincaré filter—to full measurements can lead to the discarding of basically valid data due to temporary irregularities in heart rhythm. Furthermore, variability in the length of the measurements can lead to a bias in HRV parameter values (39). Therefore, we cut the measurements into snippets of 30 min and applied the following filters to these chunks. Only data files with at least 75% valid chunks were passed on by the Poincaré filter to the next step. In the HRV filter, the mean of all chunks was calculated for every parameter, respectively, before ranking the measurements.

##### 2.2.2.2. Poincaré Filter

**Figure 1:**
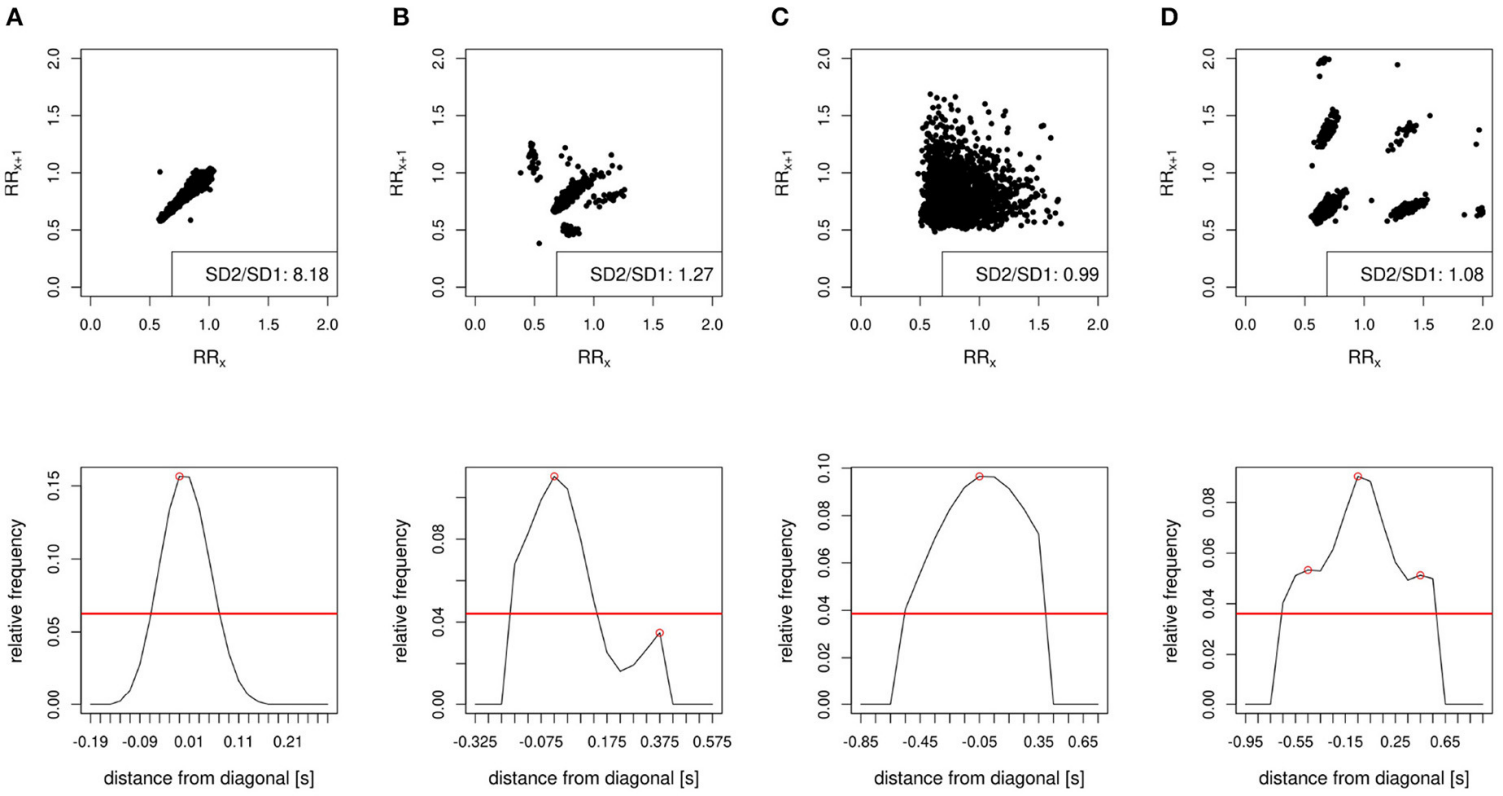
Lorenz plot patterns and their corresponding smoothed histograms of SD1. The plots show 30-min chunks of different measurements that were used in the analysis as an example of different Lorenz plot patterns. The Poincaré plots in the top show a **(A)** comet, **(B)** side lobe pattern, **(C)** fan pattern, and **(D)** island pattern. The corresponding plots below show the histograms after smoothing. The lobe, fan, and island patterns have an SD2/SD1 ratio of less than 1.5 and would, therefore, be excluded. The histogram of the island patterns includes more than one local maximum, which is another criterion for exclusion. Data: **(A)** *nsr008* from *NSR2DB*, **(B)** *nsr018* from *NSR2DB*, **(C)** *01* from *LTAFDB*, and **(D)** *f018* from *CRISDB*.

As a next step, we used a filter that quantifies the data distribution within a Poincaré plot: this non-linear method of HRV analysis plots data points of a time series against their respective successors to visualize the beat-to-beat variability of RR intervals. Any pathology that affects the length of RR-intervals causes distinct patterns in the Poincaré plots. These patterns have been systematically analyzed and categorized by Esperer et al. (40) and were called lorenz plot patterns. Plots from persons with sinus rhythm show so-called “comets” or “torpedos,” which are shaped as long cones or ellipses, respectively (Refer to Figure 1A). Other lorenz plot patterns are:

- “island” patterns consisting of four or nine roundly shaped clusters that are connected to atrial tachycardia or atrial flutter, both with the atrioventricular block (Figure 1D).
- “fan” patterns which look like broader spread torpedos or triangles and occur in persons with atrial fibrillation or multifocal atrial tachycardia (Figure 1C).
- “lobe” patterns consisting of one central and several eccentric clusters which occur due to frequent VPCs or atrial premature contractions (Figure 1B).

For our analysis, we focused on filtering out Poincaré plots with island and fan patterns since they are specific for different kinds of atrial arrhythmia and atrioventricular block. This leaves torpedos, comets, and lobe patterns that show sinus rhythm or possible VPCs. Since high-frequent VPCs are an indicator for high risk (41–43), we focused on plots that show mostly comets and torpedos. The chunk-wise analysis of the plots left enough VPCs in the resulting data. In contrast to most other shapes, torpedos and comets consist of just one evenly shaped cluster. We use this fact for two conditions of our filter: First, we projected all data points onto the axis perpendicular to the line of identity and analyzed their distribution. After taking the logarithm and smoothing, histograms with more than one extremum exceeding 40% of the maximum were excluded to rule out strong side lobe and island patterns (refer to Figure 1). Second, we calculated the SD of the projected points which is the HRV parameter SD1. Analogously, we calculated SD2 from the diagonal. The ratio of SD1 with SD2 had to exceed 1.5 since a lower ratio proved to be indicative of broader spread patterns like islands or fans. This cut-off was deduced from Esperer et al. (40), who showed that especially fans have a ratio of the length and the width of the central cluster of less than 1. We used SD1 and SD2 here since they are more commonly known and the exact methodology to create a cluster has not been described in the article.

##### 2.2.2.3. HRV Filter

The last filtering step is based on the HRV time and frequency domain of the data. We calculated the following HRV parameters with the RHRV package: SD of the averages of all normal sinus rhythm intervals (SDNN), SD of the averages of all normal sinus rhythm intervals in any 5 min segments (SDANN), triangular index, i.e., the total number of all normal sinus rhythm intervals divided by the maximum of the interval frequency distribution, square root of the mean of the squared successive differences between adjacent RR intervals (RMSSD), very low frequency power, low frequency power, high frequency power, and the ratio of low and high frequency power. For every parameter, all files were ranked for their HRV values, respectively: Files with a value that exceeded three times the interquartile range, i.e. the difference between the upper and lower quartiles (IQR), were considered to be outliers. If their values were greater or less than the median±3·IQR they were given the penalty score “–1.” For all HRV parameters, high values were assumed to be better, only for triangular index lower values were scored higher. Accordingly, the files were sorted by their HRV value and the best 20% received the score “1,” while the remaining received “0.” After this scoring process for every HRV parameter, the scores were summed up for every file, leading to possible scores from –8 (all parameter values are outliers) to 8 (all parameter values are in the top for their respective parameter). On the basis of the scores, the highest ranking 20% of the files were used to create the stVPCS.

Heart rate turbulence of all top ranking files was calculated with the *RHRT* package. All HRT calculations were done with the default settings of the *RHRT* package except for “numPostRRs” (#TSRR) for which we used 20 intervals because the longer range is the maximum of commonly used #TSRR and provided more intervals for later comparisons. For each file, the averaged VPCS was used as the basis to calculate an overall averaged VPCS (stVPCS).

#### 2.2.3. Comparing VPCSs Based on TT

##### 2.2.3.1. HRT Values

We calculated the HRT parameter values of every file in our databases with the default settings of the *RHRT* package except “numPostRRs” for which we used 30 intervals to ensure a wide range of possible TT values.

##### 2.2.3.2. DTW With stVPCS

We extracted the RR intervals in a VPCS following the compI (postRRs) of every averaged VPCS, grouped them based on their respective TT, and calculated an averaged postRRs sequence for every TT. For the next step of matching the postRRs of the stVPCS to every averaged postRRs sequence *via* DTW, we tested two methods: First, we matched the standard sequence dynamically to the averaged sequences with the default step pattern “symmetric2” of the *dtw* function. Second, we removed the leading intervals of the standard sequence before the TT to receive a sequence that only consists of the intervals that shape the TS and all following intervals. The averaged sequences were cut accordingly and shortened to fit the standard sequence. The standard sequence was matched to all averaged sequences index by index with the *dtw* step pattern *rigid*.

##### 2.2.3.3. Intra-Subject Variability of TT

As a measure of the variability of TT within a file, we calculated the SD of TT (TTSD) and the Pearson correlation coefficient for TT and TTSD.

## 3. Results

### 3.1. Comparing Data With #TSRR 15 and 20

The number of files that included enough VPCs to calculate HRT was similar with #TSRR 15 (870 files) and #TSRR 20 (862). Similarly, the number of files sorted in different HRT classes were similar with #TSRR 15 and 20 for HRT0-2 (HRT0 402 vs. 404, HRT1 139 vs. 130, and NR 321 vs. 328) as well as HRTA-C (HRTA 396 vs. 385, HRTB 144 vs. 127, and NR 322 vs. 350). Of the 862 files from which HRT parameters could be calculated in both analyzes, 43 and 55 files were differently classified into HRT classes HRT0-2 and HRTA-C, respectively (refer to Tables 2, 3).

**Table 2:** HRT0-2 classes of files before and after changing #TSRR from 15 (columns) to 20 (rows) during HRT assessment.

	**TSRR15**	***HRT0***	***HRT1***	***HRT2***	***NR***
**TSRR20**
HRT0		386	15	0	3
HRT1		9	118	0	3
HRT2		0	0	0	0
NR		7	6	0	315

*Of the 682 files that could be calculated in both TSRR15 and TSRR20, 43 files are classified differently. NR, not reliable*.

**Table 3:** HRTA-C classes of files before and after changing #TSRR from 15 (columns) to 20 (rows) during HRT assessment.

	**TSRR15**	***HRTA***	***HRTB***	***HRTC***	***NR***
**TSRR20**
HRTA		372	11	0	2
HRTB		6	118	0	3
HRTC		0	0	0	0
NR		18	15	0	317

*Of the 682 files that could be calculated in both TSRR15 and TSRR20, 55 files are classified differently. Most of the files that could be classified with #TSRR 15 are marked not reliable (NR) with #TSRR 20. NR, not reliable*.

When comparing the HRT parameters of TSRR15, TSRR20, and TSRR15∩ (data in TSRR20 recalculated with #TSRR 15), the most influenced parameter is TT with 5.47 ± 2.38 (TSRR15∩) and 5.75 ± 3 (TSRR20) (refer to Table 4). The TO values of TSRR15∩ and TSRR20 were identical, while the mean difference of the TS and TT values were 0.06 (CI 0.03 to 0.09, *p* = 4.9·10^−4^) and 0.4 (CI 0.28–0.53, *p* = 2.9·10^−10^), respectively. The most differing values of the unpaired *t*-tests were the TT values of TSRR15 and TSRR20 with CI –0.5 to 0.03 and *p* = 0.08. The *p*-values of all other unpaired *t*-tests ranged from 0.71 to 0.99 with differences of the arithmetic means between 0.001 and 0.043. A noticeable difference is the high number of TT values that were NR with #TSRR 20 (79) compared to both #TSRR 15 analyzes (9 and 6, respectively).

**Table 4:** Heart rate turbulence parameters calculated with different #TSRR.

	**TO**		**TS**		**TT**	
	**Mean ±SD**	**NR**	**Mean ±SD**	**NR**	**Mean ±SD**	**NR**
TSRR20	−2.19 ±1.51	327	4.39 ± 4.43	8	5.75 ± 3	79
TSRR15	−2.19 ±1.51	327	4.39 ± 4.41	20	5.25 ± 2.41	9
TSRR15∩	−2.19 ±1.51	327	4.39 ± 4.43	19	5.47 ± 2.38	6

*Calculations were done with 1) #TSRR 20 on all files, 2) #TSRR 15 on all files, and 3) #TSRR 15 on the intersection (∩) of the files and ventricular premature contractions (VPCs) of TSRR15 and TSRR20*.

### 3.2. Creating a stVPCS

Of the 1,080 annotation files included in the analysis, 70 files were shorter than 20 h and 1 file longer than 28 h. Thus, they were excluded.

Of the 1,009 remaining files, 652 were removed through the Poincaré filter, leaving 357 files.

After HRV parameter calculation and averaging 33 of the files contained at least one outlier. The median score of the files was 1 with a minimum of –7 and a maximum of 7.

**Figure 2:**
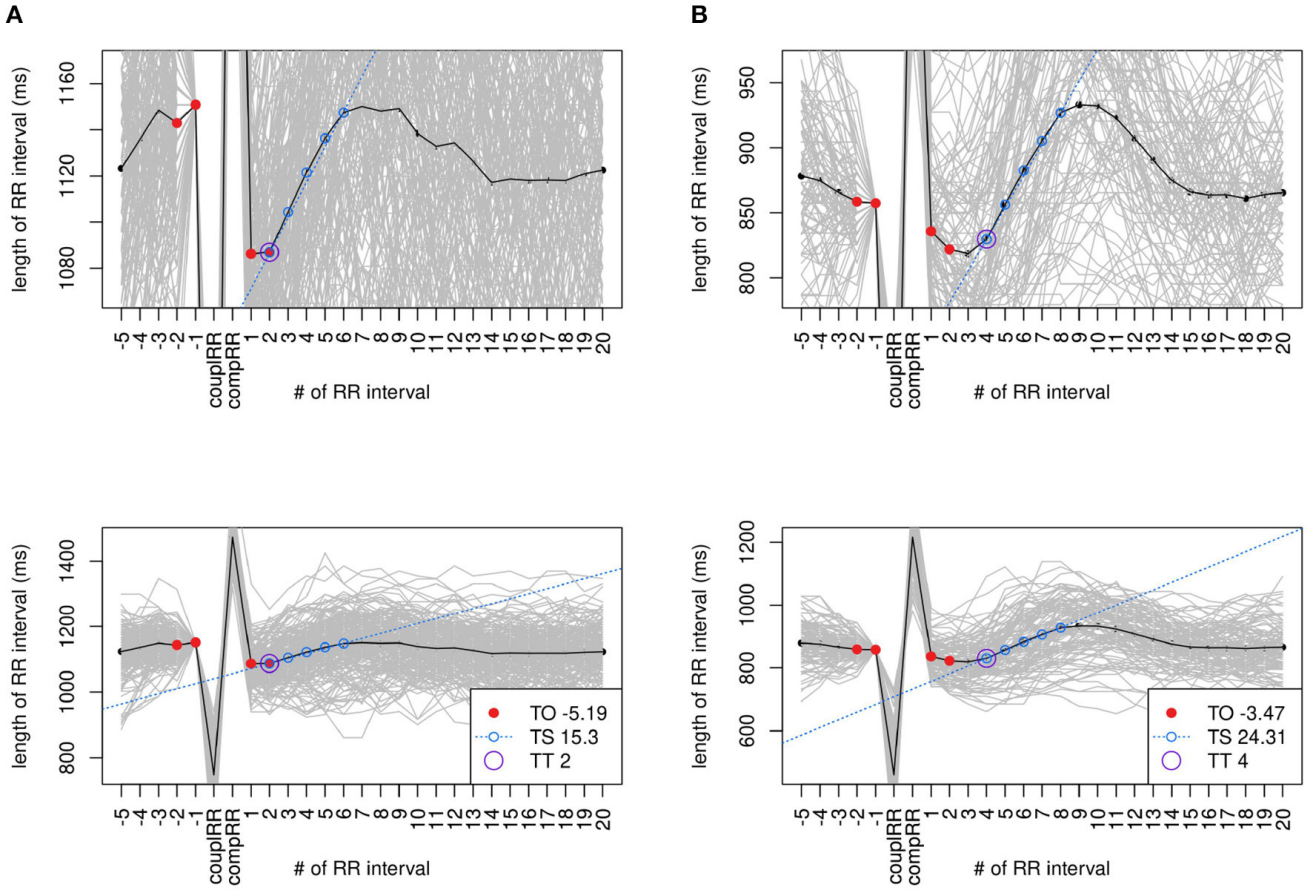
Standard VPCS (stVPCS) files with distinct heart rate turbulence (HRT). Exemplary tachograms of two files used to calculate the stVPCS that show distinct HRT. Both files are in class HRTA. The upper row shows a zoomed in tachogram, the row below the respective tachogram zoomed out. Data: **(A)** *e145a* from *CRISDB* and **(B)** *nsr010* from *NSR2DB*.

**Figure 3:**
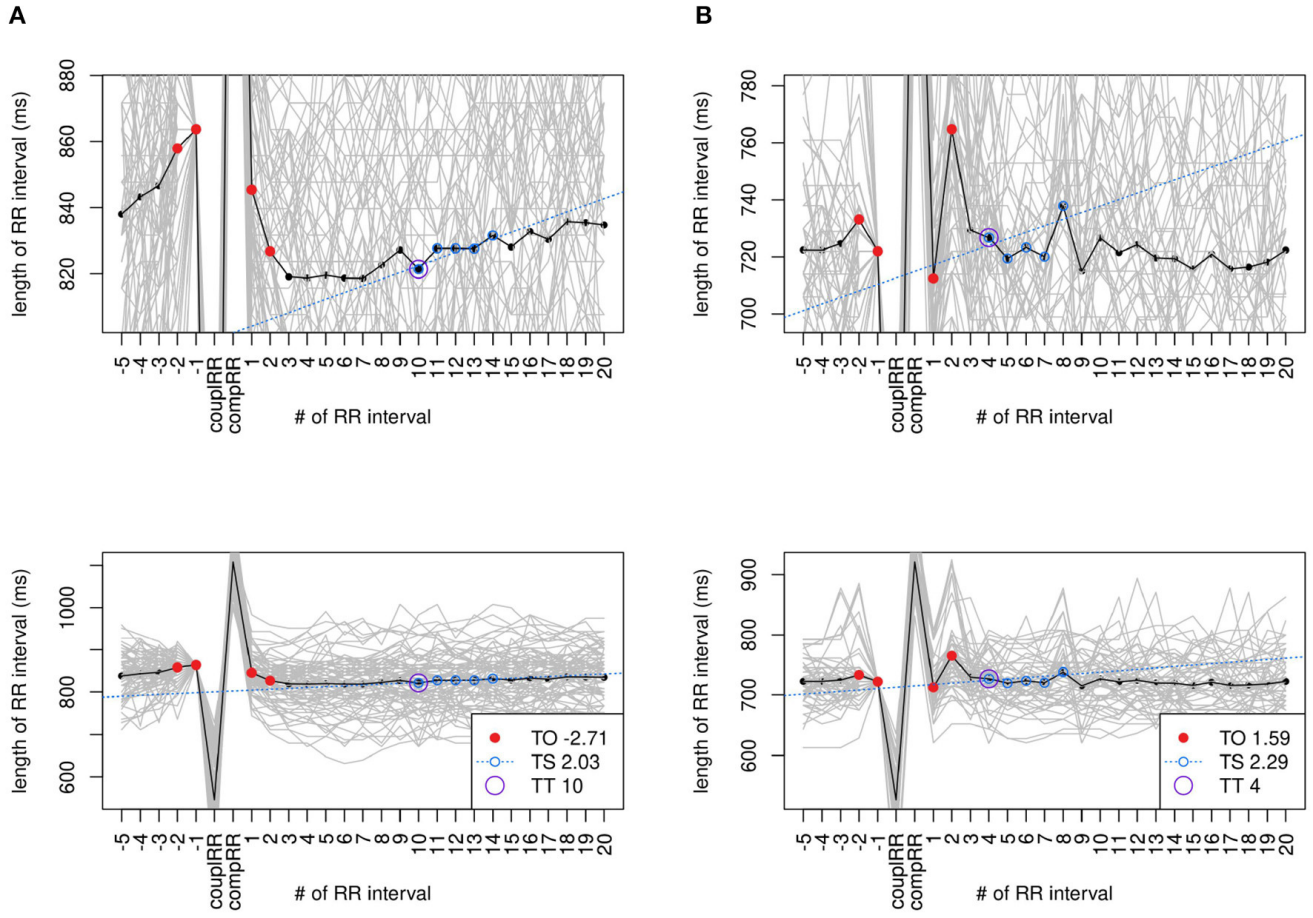
stVPCS files without distinct HRT. Exemplary tachograms of two files used to calculate the stVPCS that do not show distinct HRT. Both files are in class HRTB. The upper row shows a zoomed in tachogram, the row below the respective tachogram zoomed out. Data: **(A)** *s20491* from *LTSTDB* and **(B)** *chf202* from *CHF2DB*.

From the best 20% (71 files), HRT was calculated. In 24 files, no or too few valid VPCSs could be found. While most of the remaining 47 files showed a distinct HRT pattern (refer to Figure 2), some did not (refer to Figure 3). Table 5 shows a detailed overview of the number of filtered files broken down by databases.

**Table 5:** Overview of the number of files remaining after every analysis step sorted by their databases.

**DB**	**Input**	**Length**	**Poincaré**	**HRV**	**HRT**
CHFDB	15	0	0	0	0
CHF2DB	29	26	9	2	1
CRISDB	762	731	210	38	36
excluded	2	2	1	0	0
LTAFDB	84	76	11	3	1
LTDB	7	6	0	0	0
LTSTDB	86	81	54	13	7
NSRDB	18	17	17	6	0
NSR2DB	54	54	52	9	2
SDDB	23	16	3	0	0
Sum	1,080	1,009	357	71	47

*The different steps are Input (before any filtering), Length (after filtered for measurement length), Poincaré (after Poincaré filter), HRV (after HRV filter), and HRT (files with enough valid VPCSs to calculate HRT). DB, database*.

After classification, 41 of the files used for the stVPCS had HRT class HRT0, 5 files had HRT1, and 1 file HRT2. Of these files, 7 (2 HRT0, 4 HRT1, 1 HRT2) are marked as not reliable by the *RHRT* package. When adding TT to the classification, 40 files had HRT class HRTA and 7 files HRTB, whereas the classification from 10 files (4 of HRTA, 6 of HRTB) are marked as unreliable.

**Figure 4:**
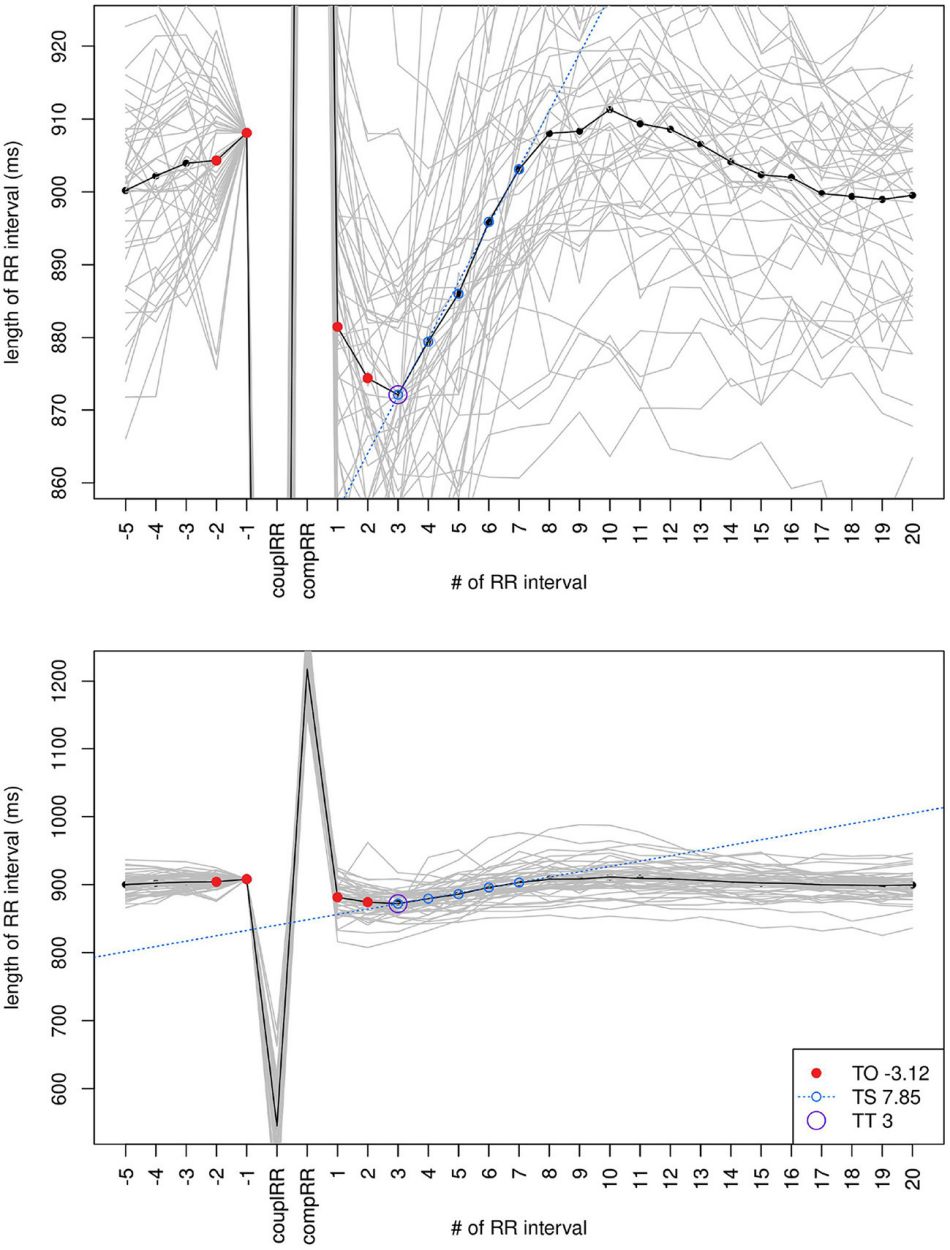
The stVPCS calculated from 47 files that matched all filter criteria and had the best HRV parameters.

Because stVPCS should be used for comparison as the ideal HRT shape, it is important that it shows a pronounced reaction to the VPC and low risk HRT parameters. The sequence averaged from all 47 VPCSs showed a distinct HRT pattern (refer to Figure 4 with TO = 3.12%, TS = 7.85 ms/RR, and TT = 3. Therefore, it falls in the lowest risk categories HRT0 and HRTA. The parameter nTS could not be calculated for stVPCS because RMSSD needs to be calculated from a respective long-term measurement, which is not applicable for the averaged VPCS.

### 3.3. Comparing VPCSs Based on TT

#### 3.3.1. HRT Values

**Figure 5:**
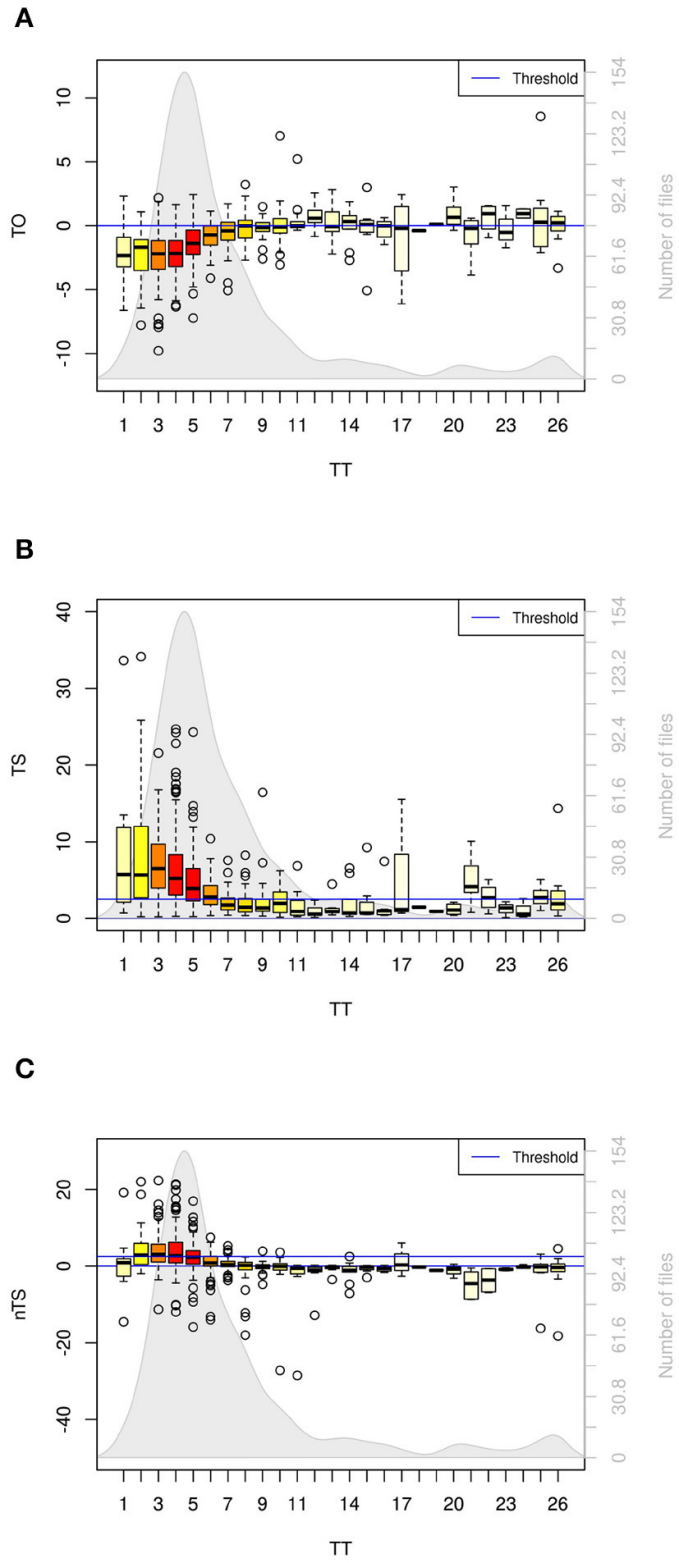
Averaged HRT parameter values of all files grouped by the respective turbulence timing (TT) value. The color of the boxes and the gray background graph illustrate the number of files in the different groups. The common threshold of TO (0%) **(A)** and TS (2.5 ms/RR) **(B)** is represented as a blue line. The common cut-off of TS is used for nTS, because no threshold for nTS has been established yet **(C)**. The median of the groups with low TT (TT ≤ 10 for TO and TT ≤ 6 for TS) lie on the low-risk side of the threshold. For nTS, only the medians of the groups with a TT of 2–4 lie above the threshold.

The HRT parameter values of all files sorted by TT can be seen in Figure 5 and in the Supplementary Table 1 in more detail. Half of the files have a TT between 4 and 8. The median TS is above the threshold of 2.5 ms/RR for TT values 6 and lower and under the threshold for most higher TT values. For high TT values, the median of TS varies, whereas the number of files in these groups is considerably lower. Unequal distribution is noticeable since the groups with more than 20 files (TT of 2 to 10) include 82% of all files. Analogously to TS, the median of TO is below the threshold for low TT values (1 to 11) and varies with increasing TT.

The parameter values of nTS worsen clearly and the pattern changes compared to the TS values: Only the medians of nTS from a TT of 2–4 still lie above the threshold. The nTS medians of all other TT values including 1 lie below the threshold. For many TT values, no file has an nTS value that exceeds the threshold.

#### 3.3.2. DTW With stVPCS

**Figure 6:**
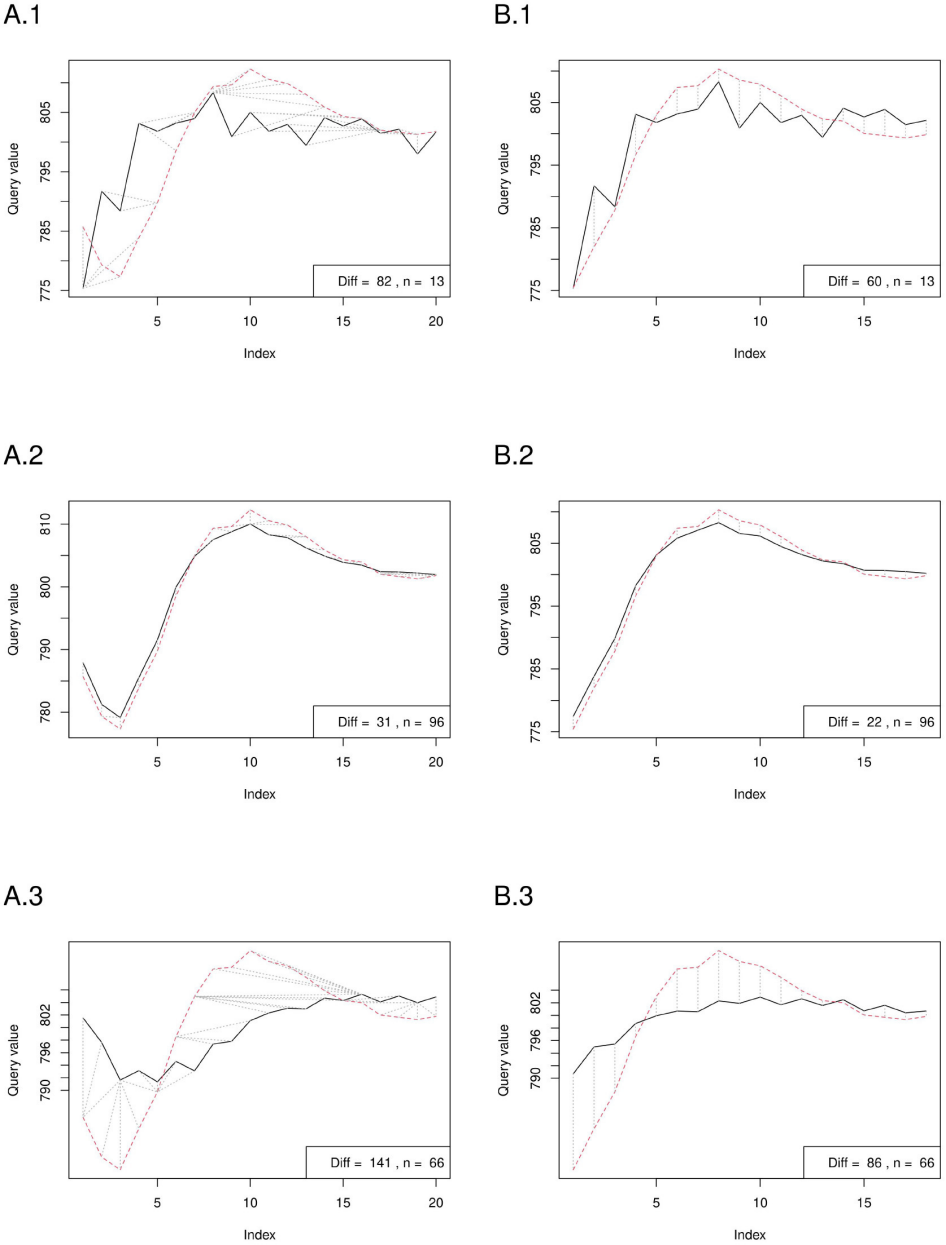
Dynamic time warping (DTW) analysis of exemplary postRRs grouped by their respective TT. The left side **(A)** shows the analysis of the stVPCS with the full postRRs sequences, the right side **(B)** with the cut sequences. From up to down the plots show the comparisons for TT 1 (1), 3 (2), and 7 (3). The full averaged sequence of TT 1 **(A.1)** lacks the initial bend and shows an immediate IL incline. The sequence of TT = 3 fits the stVPCS the best, both in the full **(A.2)** and in the cut version **(B.2)**.

**Figure 7:**
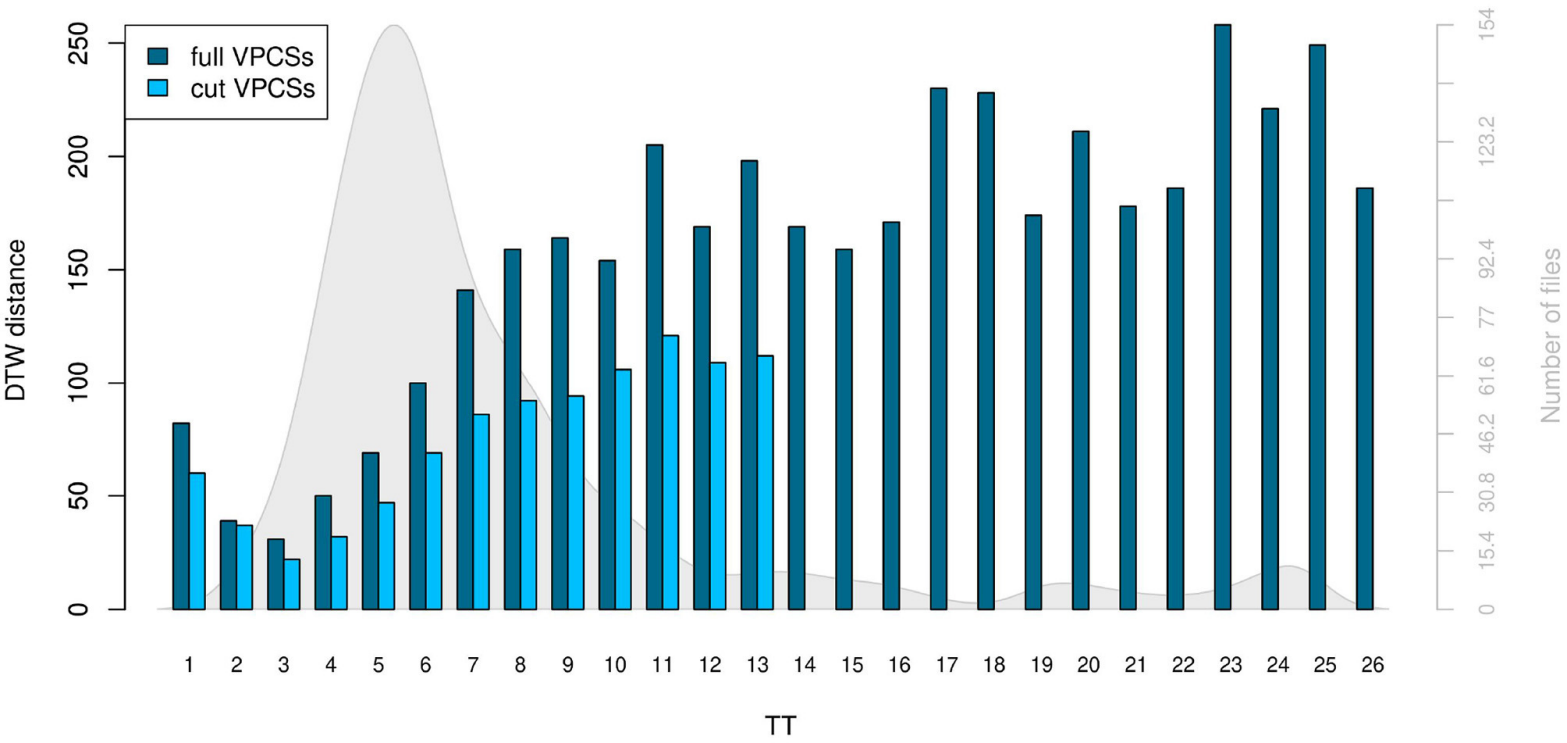
Distances calculated by DTW. The differences between stVPCS and the averaged VPCSs of all files grouped by their respective TT are shown in dark blue (full sequences) and light blue (cut sequences). The distances of the cut sequences are lower than the distances of the full sequences. In both TT 3 has the lowest distance to the stVPCS. The gray background graph and the corresponding axis on the right illustrate the number of files in the different groups.

The results of the DTW analysis are shown in Figures 6, 7. The plots for all TT values can be found in the Supplementary Figures 2 and 3. The averaged VPCS that matched the stVPCS the best was TT 3. Apart from TT 1, with rising TT, the difference between VPCS and stVPCS increased. The averaged VPCS with TT 1 lacked the characteristic delayed IL decrease but showed an immediate IL rise followed only by a shallow IL decline.

Analogously to the comparison with the full sequences, the averaged VPCS with TT 3 matched the best with the stVPCS after cutting. The difference of the VPCS of TT 1 to the best sequence is similar to the analysis without cutting (full VPCSs: Diff^TT1^ 82, Diff^TT3^ 31; with cutting: Diff^TT1^ 60, Diff^TT3^ 22). The sequences with TT 2 to 4 considerably line with the stVPCS, while the sequences flatten out continuously with rising TT.

#### 3.3.3. Intra-Subject Variability of TT

**Figure 8:**
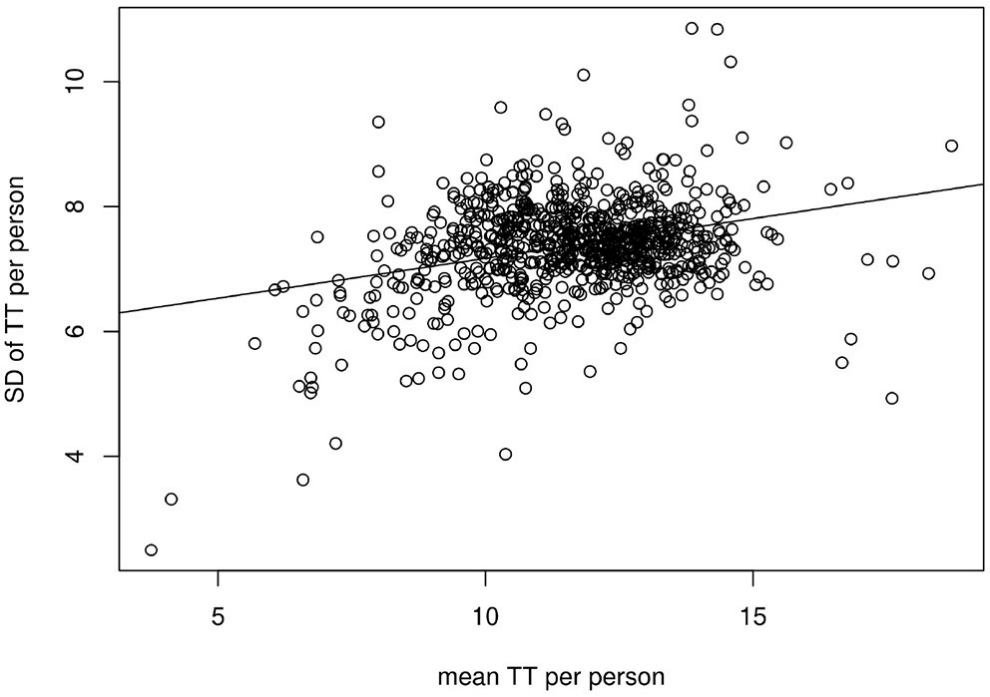
Linear regression analysis of TT and its respective TTSD of all files included in the analysis.

The TT and TTSD within a file were significantly correlated (ρ = 0.26, *p* < 0.005, refer to Figure 8).

## 4. Discussion

### 4.1. Differences in Classification

In our analysis of 1,080 files, only 8 could additionally be classified when using a lower #TSRR. Furthermore, there were 43 and 55 files that changed classification due to #TSRR in the classification systems HRT0-2 and HRTA-C, respectively. The switches were both within HRT classes as well as between an HRT class and NR. Interestingly, a high number of these files switched from an HRT class when calculated with #TSRR 15 to NR with #TSRR 20, meaning that a higher #TSRR leads to more variability in the data.

The same can be seen for TT values, where a higher amount of values was NR with #TSRR 20. This can be explained by the majority of the files with not reliable TT values showing a very shallow tachogram in visual analysis. With no distinct IL increase, random fluctuations have a stronger influence on the location of the steepest slope, thus increasing the variability of TT. Furthermore, longer VPCSs lead to a higher number of possible TT values and therefore higher variability. This high variability combined with a lower number of VPCSs results in non-significant results in the reliability check and, thus, a higher number of files with NR TT.

The only HRT parameter with distinctly differing values is TT which is to be expected with higher #TSRR. This leads to the differences in classification being marginal with less than 1% more classifiable files and 5–6% files changing the resulting classes. However, in clinical settings, even small numbers of patients that cannot be classified or are differently classified based on methodological variances are unfavorable-especially if this could be avoided by uniformly adjusting one parameter.

### 4.2. stVPCS

The stVPCS received through our pipeline shows a distinct HRT pattern with the HRT classes of HRT0 and HRTA, which imply the least possible risk. The tachogram of our stVPCS is similar to tachograms showing characteristic HRT patterns in reviews (16, 19, 22). Although the databases used to consist of files from subjects with severe diseases, with the filtering pipeline, we were able to find a set of files without pathological abnormalities based on their sound HRT parameter values. The resulting stVPCS seemed to be a feasible approximation of a healthy HRT reaction that could be used as a template for the following analysis.

### 4.3. Random Fluctuation With High TTs

Apart from TT 1, the tachograms with low TTs showed a similar pattern to the stVPCS (check the Supplementary Data Sheet 2 for a discussion of VPCS with TT 1). With increasing TT the tachograms get more shallow meaning the reaction to the VPC becomes less distinct with increasing distance to the VPC. Especially with high TT values, the tachograms show no distinct pattern but apparently random fluctuation. This can also be seen in the mean HRT values grouped by TT. As expected, the VPCSs with a low TT show the best TS values. The same can be seen for TO. With high TT values, however, the medians for both TS and TO vary, which implies common HRV rather than HRT. Still, the number of VPCSs used to calculate the medians decreases with increasing TT, which may bias this observation.

Nevertheless, TTSD is lower with lower TT values meaning that in persons with low TT the fastest slope occurs in a narrower range. Again, a narrower range implies a steady underlying mechanism that causes turbulence within a distinct time interval while a high fluctuation of TT values within a person suggests randomness. Therefore, TTSD may possibly be used as a measurement for the reliability of TT as well as TS and nTS.

Our data suggest, that only measurements with TT 2 to approximately 6 show distinct HRT. This can be seen in the DTW plots in combination with the median values of the HRT parameters grouped by TT. We recommend visually inspecting all measurements with TT 1 or higher than 7 to ensure the validity of the HRT parameters. Admittedly, manual visual inspection introduces unpreventable human error and, thus, variability to the analyzes, which should be avoided wherever possible. Therefore, DTW may be a method to ensure a reliable reaction to the VPC by comparing the progress of the tachogram of a person to a standard tachogram established from a healthy peer group. Additionally, stVPCS could be generated for different pathological conditions, which would enable using HRT not only for risk assessment but also as part of diagnostics. Possibly, DTW could replace the original HRT parameters, because it analyzes the tachogram as a whole instead of reducing it to selective parameters that can be biased as seen in this study with TS. Using DTW for HRT analysis needs establishing the mentioned stVPCSs through a sufficiently large data set with fitting health conditions and with respect to factors influencing HRT like age or circadian rhythm (31). A similar approach has already been done by Martínez et al. (44) based on a Neyman-Pearson detector (44) that compares a VPCS to the first three functions of a Karhunen-Loève transform expansion (45, 46). Under certain circumstances, this assessment is more robust regarding noise than TO and TS and needs fewer VPCSs to reach a high probability of detecting distinct HRT (46), which shows that comparison of shape patterns as a whole instead of reducing them to restricted aspects of the curve progression offers promising risk assessment parameters.

### 4.4. Hypotheses

Our first hypothesis suggested a distinct difference in HRT values when calculated with different #TSRRs. Although we could show a difference in the number of assessable HRT values and HRT classes, the differences are not as distinct as we expected with < 1% and 5–6% affected files, respectively. However, no variable risk assessment is obstructive in clinical diagnostics, especially if the results obtained from the same person vary solely based on a difference in methodology. Consequentially, the question remains which of the commonly used #TSRR are optimal for the analysis.

Therefore, our second hypothesis tackled the question of whether high TT or TTSD values do not show actual HRT but random fluctuation. The tachograms of the files with different parameter values based on #TSRR show, that these differences are mainly based on variability due to different sets of VPCSs used for calculation instead of actual HRT at the end of the VPCSs. Furthermore, the comparison of the stVPCS with averaged VPCSs grouped by TT verifies that with increasing TT the response to the VPCs decreases considerably. The same result is implied by HRT values passing their respective thresholds to an increasing degree with rising TT.

### 4.5. Limitations

While some of the files included in the stVPCS derive from NSR2DB that is defined as subjects with “normal sinus rhythm,” the vast majority of files belong to the CRISDB, LTSTDB, LTAFDB, and CHF2DB that include ECGs of persons after myocardial infarction, with ST-segment anomalies, atrial fibrillation, and congestive heart failure, respectively. Therefore, it is probable that files were included from persons with diagnosed pathologies that are not visible in the used autonomic markers and may bias our results. It would be interesting to repeat the analysis with data from healthy subjects to examine a possible difference in the stVPCSs.

Due to the lack of meta-data in the user databases, we did not analyze any influence of medication on #TSRR. To our knowledge, a temporal change in the HRT response has not been studied so far. The focus of HRT research rather lies on the strength of the response than its delay. The same goes for any response of the baroreflex: Baroreflex sensitivity has been shown to change with antihypertensive medication (47, 48), but its temporal aspect has not been studied. Since the baroreflex response latency can be influenced through short directed intervention such as tilt or atropine administration (49), it is possible that drugs influencing the sympathovagal balance like beta-blockers can change the response delay as well. However, the temporal scale of the difference does not exceed 1 s which amounts to approximately two intervals (49) and is likely to be less with long-term medication and adapted baroreceptor sensitivity. Therefore, we expect that any medication influencing HRT does not influence our results, but this also should be investigated with appropriate data.

It is important to mention that our results allow conclusions about the behavior of the autonomic marker but not its predictive power. Since HRT is a risk marker for major adverse cardiac events, analysis without meta-data about the outcome of the studied patients can only be the first step and must be verified with appropriate clinical data.

## 5. Conclusion

We recommend using #TSRR 15 for HRT analysis. The lower number of valid intervals results in a higher amount of VPCSs that can be used in the analysis as well as discarding of intervals that show random fluctuation instead of HRT. Therefore, it leads to more reliable data.

## Data Availability Statement

Publicly available datasets were analyzed in this study. This data can be found at: https://physionet.org/content/chfdb/1.0.0/; https://physionet.org/content/chf2db/1.0.0/; https://physionet.org/content/crisdb/1.0.0/; https://physionet.org/content/excluded/1.0.0/; https://physionet.org/content/ltafdb/1.0.0/; https://physionet.org/content/ltdb/1.0.0/; https://physionet.org/content/ltstdb/1.0.0/; https://physionet.org/content/nsrdb/1.0.0/; https://physionet.org/content/nsr2db/1.0.0/; https://physionet.org/content/sddb/1.0.0.

## Ethics Statement

Ethical review and approval was not required for the study on human participants in accordance with the local legislation and institutional requirements. Written informed consent for participation was not required for this study in accordance with the national legislation and the institutional requirements.

## Author Contributions

VB conceived the project, implemented the analysis pipeline and performed all analyzes and literature research, created all figures and tables, and wrote the initial draft of the manuscript and revised it. CS provided technical advice in the implementation of the analysis pipeline. CS, GE, and AD reviewed the manuscript. AD supervised the project. All authors contributed to the article and approved the submitted version.

## Conflict of Interest

The authors declare that the research was conducted in the absence of any commercial or financial relationships that could be construed as a potential conflict of interest.

## Publisher's Note

All claims expressed in this article are solely those of the authors and do not necessarily represent those of their affiliated organizations, or those of the publisher, the editors and the reviewers. Any product that may be evaluated in this article, or claim that may be made by its manufacturer, is not guaranteed or endorsed by the publisher.

## Abbreviations

ANS: autonomic nervous system
compI: compensatory interval
couplI: coupling interval
CRAN: The Comprehensive R Archive Network
DTW: dynamic time warping
ECG: electrocardiogram
HRT: heart rate turbulence
HRV: heart rate variability
IL: interval length
IQR: interquartile range, i.e., the difference between the upper and lower quartiles
ISHNE: International Society for Holter and Noninvasive Electrocardiology
NR: not reliable
nRMSSD: RMSSD normalized for heart rate
nTS: TS normalized after (1)
numTSRR[#TSRR]: number of RR intervals in which TS is calculated
postRRs: RR intervals in a VPCS following the compI
preRRs: RR intervals in a VPCS before the couplI
refI: reference interval
RMSSD: square root of the mean of the squared successive differences between adjacent RR intervals
SD: standard deviation
SD1: SD of data points in poincare plot projected to the axis perpendicular to the line of identity
SD2: SD of data points in poincare plot projected to the line of identity
SDANN: standard deviation of the averages of all normal sinus rhythm intervals in any 5 min segments
SDNN: standard deviation of the averages of all normal sinus rhythm intervals
stVPCS: standard VPCS
TO: turbulence onset
TS: turbulence slope
TT: turbulence timing
TTSD: SD of TT
VPCS: VPC snippet, i.e., all RR intervals surrounding the VPC used for HRT calculation
VPC: ventricular premature contraction.
