# Supplementary material for: Comparability of Heart Rate Turbulence Methodology: 15 Intervals Suffice to Calculate Turbulence Slope – A Methodological Analysis Using PhysioNet Data of 1074 Patients
Source: Front Cardiovasc Med. 2022 Apr 6;9:793535. doi: 10.3389/fcvm.2022.793535 (PMC9019151; doi:10.3389/fcvm.2022.793535)
Supplement: Supplementary file 1 [file Table_1.pdf]

Averaged HRT parameter values of all files grouped by the respective turbulence timing (TT) value.

For a visual representation of this data see the corresponding figure 5 in the paper.

**TT**	**n**	**TO**				**TS**			
		**median**	**mean**	**±**	**SD**	**median**	**mean**	**±**	**SD**
1	13	-2.34	-2.33	±	2.35	5.73	8.02	±	8.84
2	34	-1.68	-2.32	±	2.05	5.67	8.23	±	7.71
3	96	-2.2	-2.33	±	2.12	6.49	7.18	±	4.44
4	149	-2.18	-2.2	±	1.61	5.2	6.5	±	4.96
5	154	-1.39	-1.45	±	1.56	3.91	4.68	±	3.43
6	83	-0.72	-0.88	±	1.07	2.78	3.24	±	1.95
7	66	-0.4	-0.48	±	1.16	1.74	2.09	±	1.35
8	53	-0.02	-0.23	±	1.12	1.46	1.96	±	1.53
9	28	-0.14	-0.19	±	0.81	1.38	2.32	±	3.13
10	25	-0.11	0.12	±	1.85	1.96	2.25	±	1.81
11	14	-0.01	0.49	±	1.43	0.91	1.59	±	1.8
12	7	0.59	0.74	±	1.07	0.55	0.95	±	0.8
13	9	-0.07	0.22	±	1.42	0.89	1.3	±	1.23
14	10	0.32	-0.06	±	1.38	0.69	1.99	±	2.33
15	7	0.08	-0.32	±	2.42	0.68	2.27	±	3.19
16	7	-0.01	-0.29	±	0.82	0.97	1.71	±	2.54
17	4	-0.21	-1.03	±	3.67	1.16	4.63	±	7.27
18	1	-0.39	-0.39	±	NA	1.48	1.48	±	NA
19	1	0.1	0.1	±	NA	0.91	0.91	±	NA
20	8	0.66	0.88	±	1.1	1.08	1.18	±	0.7
21	6	-0.21	-0.79	±	1.68	4.14	4.9	±	3.2
22	4	0.94	0.62	±	1.16	2.69	2.75	±	1.85
23	3	-0.52	-0.23	±	1.66	1.38	1.21	±	1.05
24	4	0.95	0.95	±	0.4	0.55	0.96	±	1.09
25	8	0.26	0.8	±	3.44	2.7	2.83	±	1.3
26	15	0.21	-0.03	±	1.12	1.91	2.91	±	3.41
