# Supplementary material for: Comparability of Heart Rate Turbulence Methodology: 15 Intervals Suffice to Calculate Turbulence Slope – A Methodological Analysis Using PhysioNet Data of 1074 Patients
Source: Front Cardiovasc Med. 2022 Apr 6;9:793535. doi: 10.3389/fcvm.2022.793535 (PMC9019151; doi:10.3389/fcvm.2022.793535)
Supplement: Supplementary file 2 [file Table_2.pdf]

Differences calculated with dynamic time warping (DTW) between standard VPCS (stVPCS) and the averaged VPCS of all files grouped by their respective turbulence timing (TT).
Diff (full) gives the differences to the full sequences, *Diff (cut)* the differences to the cut sequences. Because the number of intervals of the cut stVPCS is 18 and the number of RR intervals in a VPCS following the compI (postRRs) of the sequences sorted for TT was 30, the last cut sequence that can be compared to the stVPCS has TT 13 (all following sequences would contain less intervals than the cut stVPCS).

TT	n	Diff (full)	Diff (cut)
1	13	82	60
2	34	39	37
3	96	31	22
4	149	50	32
5	154	69	47
6	83	100	69
7	66	141	86
8	53	159	92
9	28	164	94
10	25	154	106
11	14	205	121
12	7	169	109
13	9	198	112
14	10	169	–
15	7	159	–
16	7	171	–
17	4	230	–
18	1	228	–
19	1	174	–
20	8	211	–
21	6	178	–
22	4	186	–
23	3	258	–
24	4	221	–
25	8	249	–
26	15	186	–
