# Supplementary material for: Comparability of Heart Rate Turbulence Methodology: 15 Intervals Suffice to Calculate Turbulence Slope – A Methodological Analysis Using PhysioNet Data of 1074 Patients
Source: Front Cardiovasc Med. 2022 Apr 6;9:793535. doi: 10.3389/fcvm.2022.793535 (PMC9019151; doi:10.3389/fcvm.2022.793535)
Supplement: Supplementary file 3 [file Data_Sheet_1.pdf]

Detailed Methodology: HRT Assessment of RHRT

Filtering If given annotation data, *RHRT* checks the surrounding intervals of the beats marked as ventricular premature contractions (VPCs). If they match the filtering rules, the snippets are stored and heart rate turbulence (HRT) parameters are calculated. If not stated otherwise, the filtering and calculation rules follow the suggested methodology of (*1*):

The reference interval (refI) is calculated as the arithmetic mean of the five intervals preceding the coupling interval (couplI). It is used as an approximation of the mean interval length (IL) of the VPC snippet, i.e. all RR intervals surrounding the VPC used for HRT calculation (VPCS) in order to exclude biased intervals from the HRT assessment. A valid VPCS consisting of 5 intervals before the couplI and 15 intervals after the compensatory interval (compI) must match all following filter rules to be included in the calculation:

- apart from the VPC, all beats must be normal sinus rhythm contractions
- the couplI must have a maximal length of 80 % of the refI
- the compI must have a minimal length of 120 % of the refI
- all regular intervals (meaning RR intervals in a VPCS before the couplI (preRRs) and RR intervals in a VPCS following the compI (postRRs)) must
  - be between 300 ms and 2000 ms
  - not differ more than 20 % from the refI
  - not differ more than 200 ms from the preceding interval

Parameter Calculation If a measurement includes 5 or more valid VPCs its HRT can be determined as follows: turbulence onset (TO) is calculated from the two intervals before and after the couplIs and compI, respectively, with

$$((RR_1 + RR_2) - (RR_{-1} + RR_{-2}))/(RR_{-1} + RR_{-2}) * 100[\%] \tag{1}$$

turbulence slope (TS) is calculated as the steepest slope of any consecutive 5 intervals within the postRRs. The default number of postRRs which is equal to number of RR intervals in which TS is calculated (#TSRR) in *RHRT* is 15. While TO is calculated from every VPCS directly and averaged as an arithmetic mean afterwards, TS is assessed from an averaged tachogram. The averaged tachogram consists of arithmetic means of the intervals with the same respective indices. turbulence timing (TT) is the index of the first interval used to calculate TS.

Lastly, nTS is the TS normalised after the method suggested in (*2*): TS is first normalised to an heart rate of 75 bpm (800 ms IL) before using the following formula:

$$nTS = TS - (0.02475 \cdot k^{0.9449} \frac{nRMSSD}{\sqrt{\#VPCSs}}) \tag{2}$$

Here, k is #TSRR and nRMSSD is the square root of the mean of the squared successive differences between adjacent RR intervals (RMSSD) normalised for the heart rate of the whole measurement.

Classification *RHRT* can return common HRT classes of the given data. With the HRT categorisation systems HRT0-2 (based on TO and TS) and HRTA-C (based on TO, TS and TT) measurements can be classified into low and high risk groups based on the implication of their parameter values in respect to established thresholds. HRT0-2 includes HRT0 (low risk: TO is below and TS above their respective thresholds), HRT1 (intermediate risk: one of the parameter values passes its threshold) and HRT2 (high risk: both parameter values implying high risk). When including TT the classification system HRTA-C can be used consisting of HRTA (low risk: all parameter values with low risk), HRTB (intermediate risk: at least one parameter value with high risk) and HRTC (high risk: all parameter values exceed their thresholds). The default thresholds used for the HRT parameters are 0 % (TO), 2.5 ms/RR (TS) and index 10 (TT). Per default *RHRT* uses only measurements that have at least 5 VPCSs that fit the filtering criteria and discards files with less suitable VPCSs.

RHRT allows to check the reliability of averaged parameter values. The HRT values calculated separately from the underlying VPCSs of one parameter are used for a one-sided t-test against the respective threshold of the parameter. If the result exceeds 0.05 the averaged parameter value is marked as not reliable (NR). This ensures that parameter values based on a low number of VPCSs, with high variability and close to the threshold are not used for classification. Therefore, only parameter values having a high possibility of being accurate are used.

A statistical hypothesis test should never replace a judicious examination of data. Here, we use a t-test with a strict cut-off to categorise analyses that are intrinsically non-separable. Because of missing semantic information about the data and with high uncertainty coming with a low number of data points, a trustworthy declaration of the true parameter values is impossible. However, we use this test to add a bit of certainty to an analysis that neglects whether the calculated parameter value or classification is based on a solid, non-ambiguous data set. In this study, the reliability check serves to discard data that has a high probability of false parameter values. In clinical practice, it should be used as one of many factors to determine the reliability of the calculated HRT results. For more in-

formation about the difficulty of using a strict dichotomy, especially based on the p-value, see the respective discussion (*3*, *4*).

References

1. V. Blesius, C. Schölzel, G. Ernst, A. Dominik, HRT assessment reviewed: a systematic review of heart rate turbulence methodology. *Physiological Measurement* **41**, 08TR01 (2020).

2. A. P. Hallstrom *et al.*, Structural relationships between measures based on heart beat intervals: potential for improved risk assessment. *IEEE transactions on bio-medical engineering* **51**, 1414–1420 (2004).

3. V. Amrhein, S. Greenland, B. McShane, Scientists rise up against statistical significance. *Nature* **567**, 305–307 (2019).

4. R. L. Wasserstein, A. L. Schirm, N. A. Lazar, Moving to a World Beyond "p $<$ 0.05". *The American Statistician* **73**, 1–19 (sup1 2019).
