# Supplementary material for: Comparability of Heart Rate Turbulence Methodology: 15 Intervals Suffice to Calculate Turbulence Slope – A Methodological Analysis Using PhysioNet Data of 1074 Patients
Source: Front Cardiovasc Med. 2022 Apr 6;9:793535. doi: 10.3389/fcvm.2022.793535 (PMC9019151; doi:10.3389/fcvm.2022.793535)
Supplement: Supplementary file 4 [file Data_Sheet_2.pdf]

Further Discussion: no HRT with TT 1?

While the median values of turbulence onset (TO) and turbulence slope (TS) of all files with turbulence timing (TT) 1 imply low-risk according to their thresholds, TS normalised after Hallstrom *et al.* (nTS) falls below the threshold and the tachograms of the VPC snippet, i.e. all RR intervals surrounding the VPC used for HRT calculation (VPCS) with TT 1 both with and without cutting do not match the standard VPCS (stVPCS). The different risk implications of TS and nTS can be explained by the fact that we used the same cut-off value for both parameters. Until now no different cut-off value was proposed, but it is likely that nTS needs an adjusted cut-off value. However, there are also other factors that make the assessment of nTS difficult. The files with TT 1 tend to have a higher square root of the mean of the squared successive differences between adjacent RR intervals (RMSSD) compared to the other files with low TT values (data not shown). This affects nTS directly, because the parameter nTS takes the number of underlying VPCSs into account to compensate for the mathematical bias it brings in. Conflinctingly, while a high nTS means less risk, a high RMSSD implies less risk, but results in a lower nTS. While the importance of a normalised TS has been shown as reviewed in (*2*), the nTS proposed by (*1*) needs further investigation regarding its usability and a cut-off possibly different from TS.

Furthermore, since the number of files with TT 1 is very low (13), random fluctuations can have a high influence on the statistical analysis as can be seen on the files with high TT. Therefore, the parameter values as well as the VPCS may not be robust and should be repeated with a larger data set. Thus, our data does not provide a definitive conclusion whether TT 1 is physiologically plausible or could be related to autonomic imbalance.

References

1. A. P. Hallstrom *et al.*, Structural relationships between measures based on heart beat intervals: potential for improved risk assessment. *IEEE transactions on bio-medical engineering* **51**, 1414–1420 (2004).
2. V. Blesius, C. Schölzel, G. Ernst, A. Dominik, HRT assessment reviewed: a systematic review of heart rate turbulence methodology. *Physiological Measurement* **41**, 08TR01 (2020).
