# Supplementary material for: Comparability of Heart Rate Turbulence Methodology: 15 Intervals Suffice to Calculate Turbulence Slope – A Methodological Analysis Using PhysioNet Data of 1074 Patients
Source: Front Cardiovasc Med. 2022 Apr 6;9:793535. doi: 10.3389/fcvm.2022.793535 (PMC9019151; doi:10.3389/fcvm.2022.793535)
Supplement: Supplementary file 5 [file Image_1.pdf]

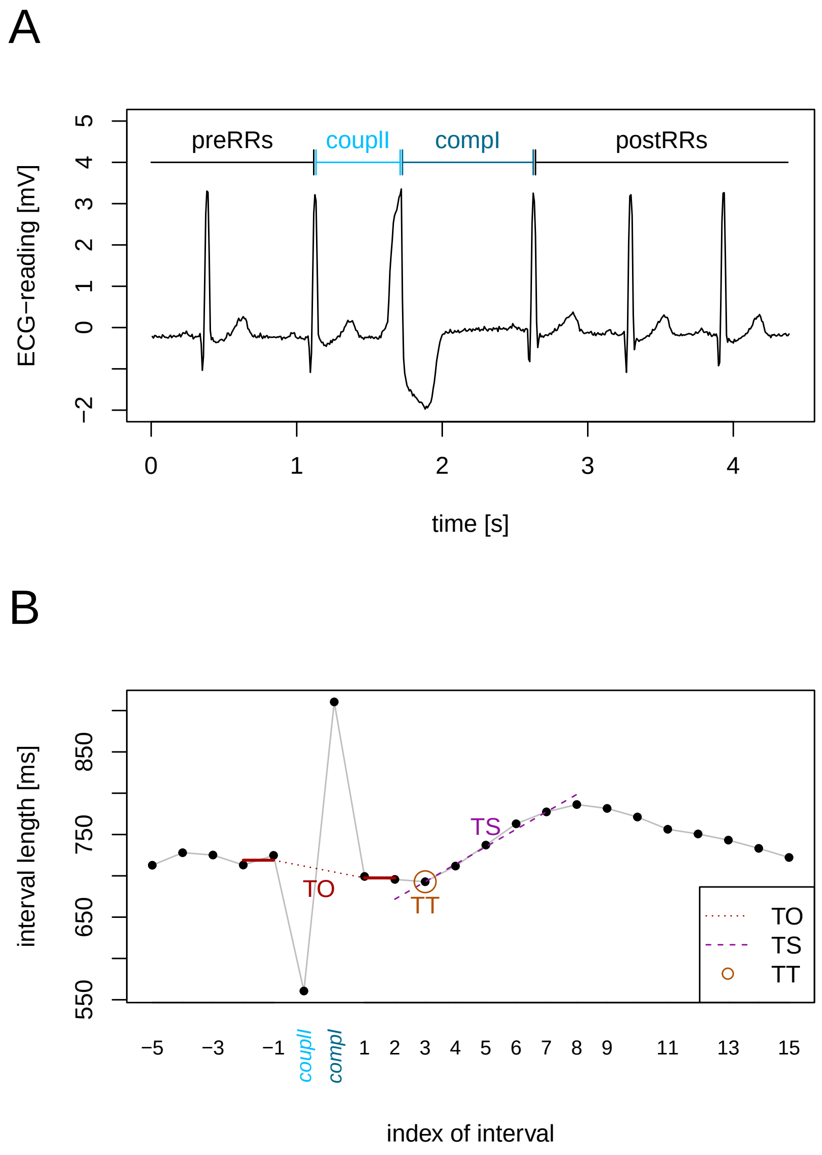

Exemplary ECG and tachogram of HRT.
(A) The shortened RR interval between the VPC and its preceding beat is called coupling interval (couplI) and is followed by the prolonged compensatory interval (compI). The RR intervals before the couplI are called preRRs, the ones after postRRs. **(B)** After the short couplI and the long compI the interval length drops slightly and increases distinctly afterwards. The HRT parameters turbulence onset (TO), turbulence slope (TS) and turbulence timing (TT) are marked in light, medium and dark green, respectively. Data: *16265* from *NSRDB*. Figures adapted from (*1*).

References

1. V. Blesius, C. Schölzel, G. Ernst, A. Dominik, HRT assessment reviewed: a systematic review of heart rate turbulence methodology. *Physiological Measurement* **41**, 08TR01 (2020).
