# Supplementary material for: Comparability of Heart Rate Turbulence Methodology: 15 Intervals Suffice to Calculate Turbulence Slope – A Methodological Analysis Using PhysioNet Data of 1074 Patients
Source: Front Cardiovasc Med. 2022 Apr 6;9:793535. doi: 10.3389/fcvm.2022.793535 (PMC9019151; doi:10.3389/fcvm.2022.793535)
Supplement: Supplementary file 6 [file Image_2.pdf]

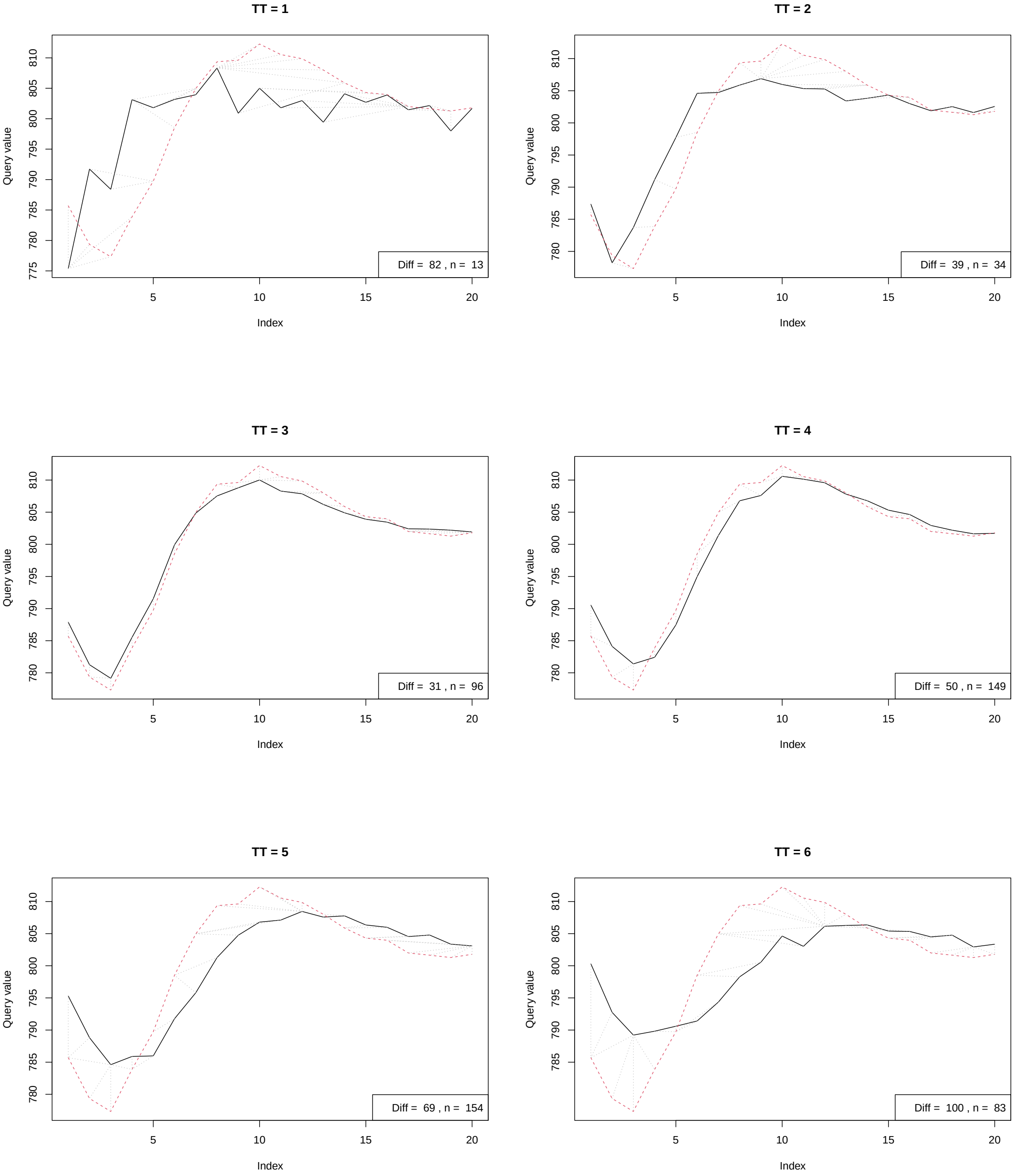

Dynamic time warping (DTW) analysis of postRRs grouped by their respective TT (1-6).

The DTW analysis compared the postRRs from the standard VPCS (stVPCS) and the averaged VPC snippet, i.e. all RR intervals surrounding the VPC used for HRT calculations (VPCSs) of all files grouped by their respective turbulence timing (TT) (1 to 6). The averaged sequence of TT = 1 lacks the initial bend and shows an immediate interval length (IL) incline. The sequence of TT = 3 fit the stVPCS the best.

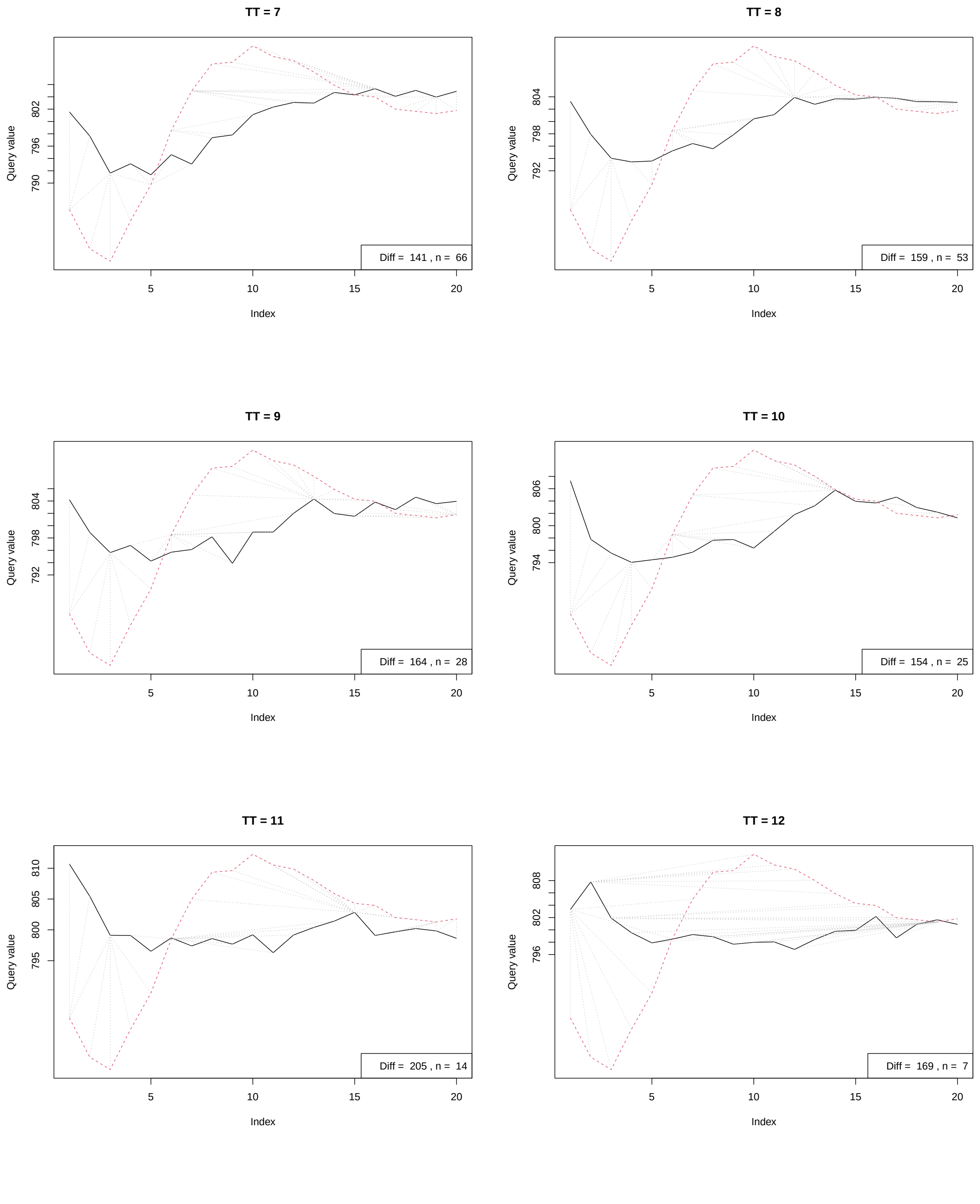

DTW analysis of postRRs grouped by their respective TT (7-12). With rising TT values the tachograms flatten increasingly.

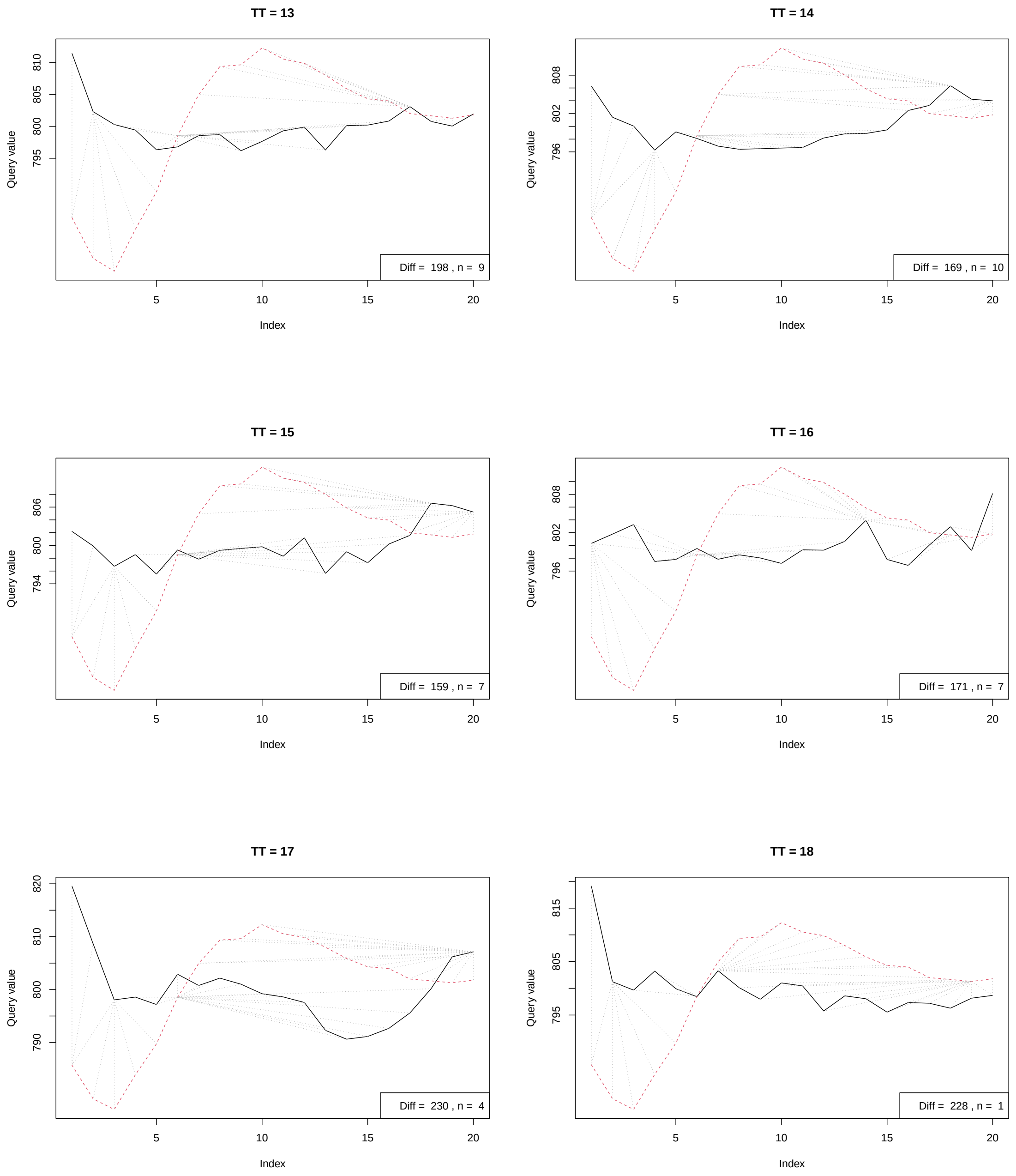

DTW analysis of postRRs grouped by their respective TT (13-18). The typical heart rate turbulence (HRT) pattern is no longer visible. The sequences are more jagged because of the low number of underlying files.

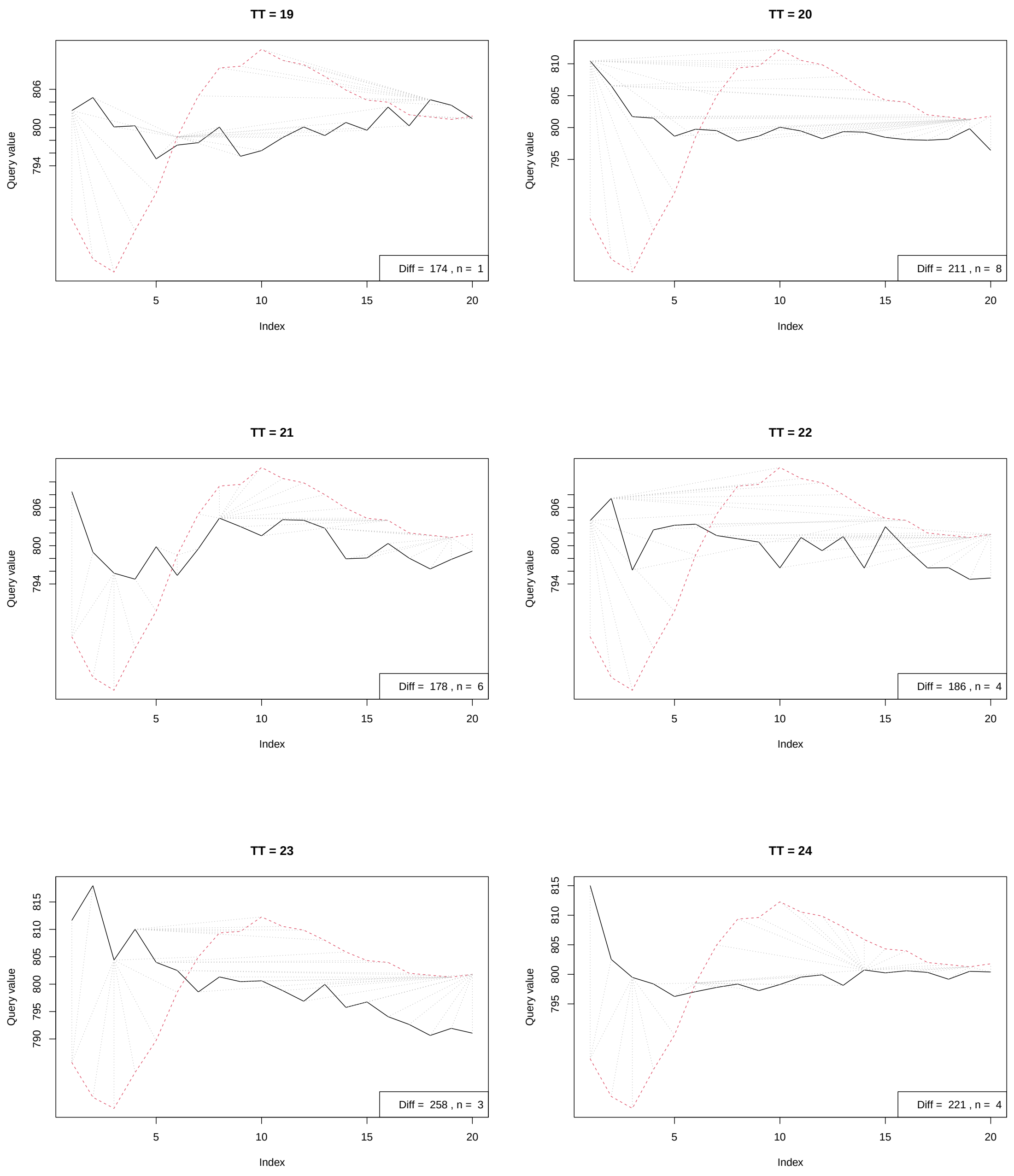

DTW analysis of postRRs grouped by their respective TT (19-24).

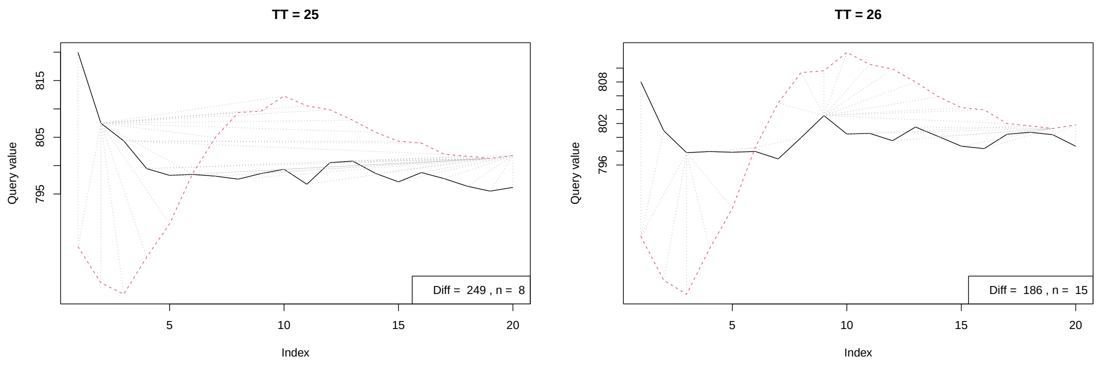

DTW analysis of postRRs grouped by their respective TT (25 & 26).
