# Supplementary material for: Comparability of Heart Rate Turbulence Methodology: 15 Intervals Suffice to Calculate Turbulence Slope – A Methodological Analysis Using PhysioNet Data of 1074 Patients
Source: Front Cardiovasc Med. 2022 Apr 6;9:793535. doi: 10.3389/fcvm.2022.793535 (PMC9019151; doi:10.3389/fcvm.2022.793535)
Supplement: Supplementary file 7 [file Image_3.pdf]

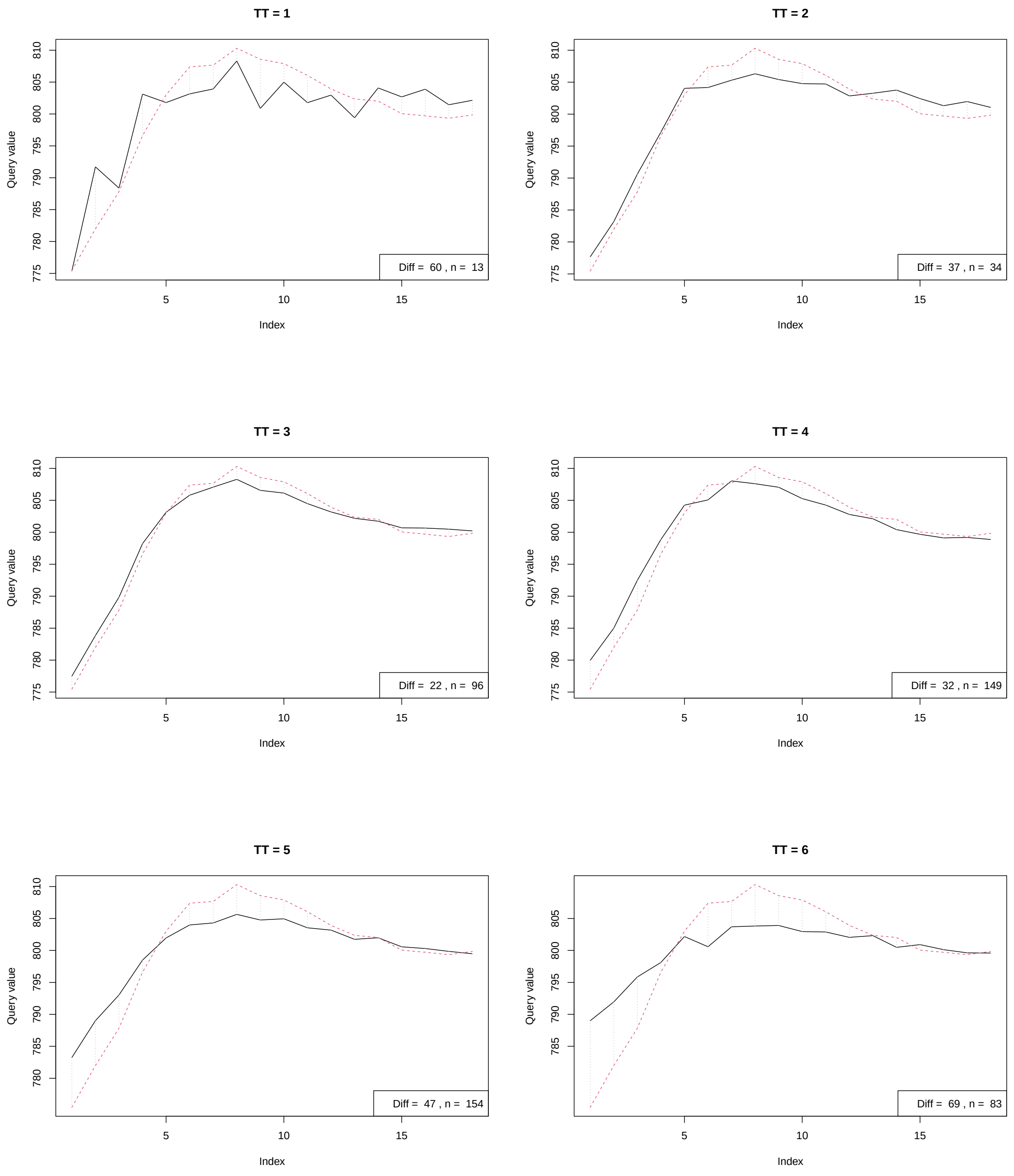

Dynamic time warping (DTW) analysis of postRRs grouped by their respective turbulence timing (TT) (1-6).

DTW analysis of the cut postRRs from the standard VPCS (stVPCS) and the averaged VPC snippet, i.e. all RR intervals surrounding the VPC used for HRT calculations (VPCSs) of all files grouped by their respective TT (1 to 6). Again, the sequence of TT = 2 fit the stVPCS the best.

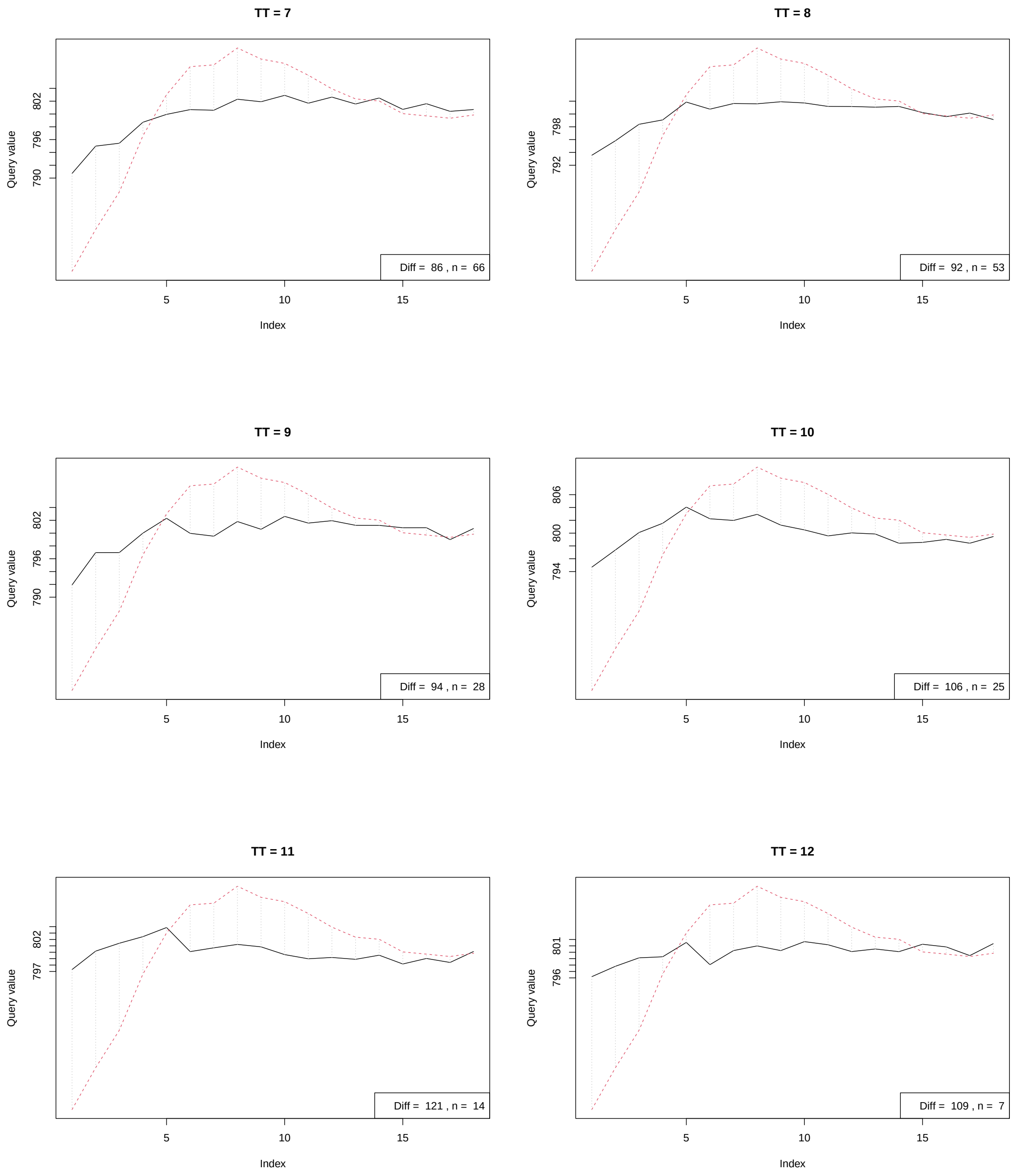

DTW analysis of postRRs grouped by their respective TT (7-12). With rising TT values the tachograms flatten increasingly and lose the interval length (IL) decline.

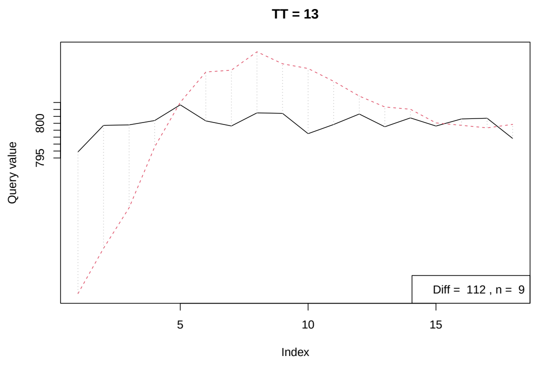

DTW analysis of postRRs grouped by their respective TT (13). The tachogram flattened compared to the tachograms with low TT and lost the IL decline.
